# Foundational ingredients of robotic gait training for people with incomplete spinal cord injury during inpatient rehabilitation (FIRST): A randomized controlled trial protocol

**DOI:** 10.1371/journal.pone.0267013

**Published:** 2022-05-10

**Authors:** Chad Swank, Alexandria Holden, Lacy McDonald, Simon Driver, Librada Callender, Monica Bennett, Seema Sikka

**Affiliations:** 1 Baylor Scott and White Research Institute, Dallas, Texas, United States of America; 2 Baylor Scott & White Institute for Rehabilitation, Dallas, Texas, United States of America; 3 Baylor Scott & White Health, Dallas, Texas, United States of America; Public Library of Science, UNITED KINGDOM

## Abstract

**Introduction:**

As technological advances allow the use of robotic exoskeleton devices with gait training, there is a critical need to establish a robotic gait training (RGT) program to meet the needs of people with spinal cord injury (SCI) during inpatient rehabilitation. The purposes of this study are to prospectively examine the efficacy of a stakeholder informed RGT program compared to usual care gait training (UC) during inpatient rehabilitation in people with incomplete SCI and compare the intensity of RGT and UC gait training during inpatient rehabilitation.

**Study design:**

128 patients with incomplete SCI admitted to our inpatient rehabilitation facility will be screened for eligibility and randomized to either the RGT or UC group. RGT sessions will use the Ekso robotic exoskeleton [class II medical device (United States FDA)]. UC sessions will use traditional gait training approaches such as manually assisted overground gait training with walkers and orthotics and body weight–supported treadmill training (BWSTT). Our primary outcome is gait function as characterized by the Walking Index for Spinal Cord Injury–II (WISCI-II). Secondary outcomes are gait speed, Spinal Cord Independence Measure (SCIM), Numeric Pain Rating Scale (NPRS), Fatigue Severity Scale (FSS), Penn Spasm Frequency Scale (PSFS), Patient Health Questionnaire-9 (PHQ-9), General Anxiety Disorder– 7 (GAD-7), International Spinal Cord Injury Quality of Life Basic Data Set, and a Qualitative Questionnaire. Assessments of primary and secondary outcomes will occur at admission and discharge from inpatient rehabilitation. General or generalized linear models will be used to analyze differences between groups for all measures.

**Clinical impact:**

Successful completion of this study will provide a usable, replicable, stakeholder informed RGT intervention for use with individuals with incomplete SCI during inpatient rehabilitation.

## Introduction

Spinal cord injury (SCI) due to trauma is estimated to affect 288,000–500,000 Americans, with about 17,700 new cases annually [1]. Despite general advances in medicine, the average life expectancy for persons with SCI has remained largely unchanged since the 1980s and are significantly lower than for persons without SCI [2, 3]. Given this disparity of life expectancy and decreased quality of life after SCI [4], gait training has the opportunity to positively impact the lives of people after SCI. Not surprisingly, recovery of walking is a primary rehabilitation goal for patients and encouraged by therapists [5, 6] due to its relationship to quality of life [7, 8]. impact on health, psychological profile [9], and social participation after SCI [10, 11].

Advances in technology are being adopted into clinical rehabilitation practice at a rapid pace. Robotic exoskeletons (wearable robots to enhance overground mobility) are a potentially viable technology that can be used for gait training among those with acute SCI. Yet, foundational evidence to support exoskeleton use during the acute phase of recovery is lacking. While robotic exoskeleton technology is not yet mature enough to produce independent community ambulation [12, 13], the technology has improved [14] and may allow for enhanced SCI rehabilitation [15].

Today four FDA-approved exoskeleton devices are commercially available that allow people to step over-ground using variable levels of assist depending on the individual’s ability [13]. The contemporary robotic exoskeleton is a rechargeable bionic device worn over the lower extremities with motorized joints that can provide externally-powered gait independent of a treadmill system [16] and allow repetitive task specific movement training [15]. The focus of this protocol was the use of an exoskeleton to enhance recovery of gait through over-ground repetitive task specific movement. This protocol paper followed the Standard Protocol Items: Recommendations for Interventional Trials (SPIRIT) checklist to report relevant clinical trial details as recommended by the EQUATOR Network (Enhancing the Quality and Transparency of Health Research).

### Objectives and aims

**Aim 1**: Develop a robotic gait training (RGT) program that meets the unique needs of people after incomplete SCI during inpatient rehabilitation.

Hypothesis 1.1: Engage key stakeholders (patients, caregivers, clinicians, researchers, industry members) in a community-based participatory research approach to develop the content and structure of the RGT program to accommodate current clinical practice guidelines and integrate emerging robotic exoskeleton technology.

**Aim 2**: Examine the efficacy of RGT compared to usual care gait training (UC) during inpatient rehabilitation in people with incomplete SCI.

Hypothesis 2.1: Patients with incomplete SCI who receive RGT will have higher walking function (Walking Index for Spinal Cord Injury–Revised (WISCI-II)) at discharge compared to UC.
Hypothesis 2.2: Patients with incomplete SCI who receive RGT will have greater improvement in secondary outcomes (gait speed, daily functional independence, pain, fatigue, spasm frequency, depressive symptoms, and quality of life) at discharge compared to UC.

**Aim 3**: Compare the intensity of RGT and UC gait training during inpatient rehabilitation. Intensity data will include (1) heart rate, (2) rate of perceived exertion (RPE), and (3) number of steps.

Hypothesis 3.1: RGT will be of significantly greater intensity with participants (1) spending more minutes per session and total minutes (over all sessions) in moderate intensity exercise (40% to 60% range of heart rate reserve), (2) reporting higher mean RPE across all sessions, and (3) completing more steps during each session and total number of steps over all sessions compared to UC.
Hypothesis 3.2: Intensity will be significantly correlated to the primary outcome (WISCI-II) for the RGT and UC groups.

## Methods

### Study design

**Fig 1:**
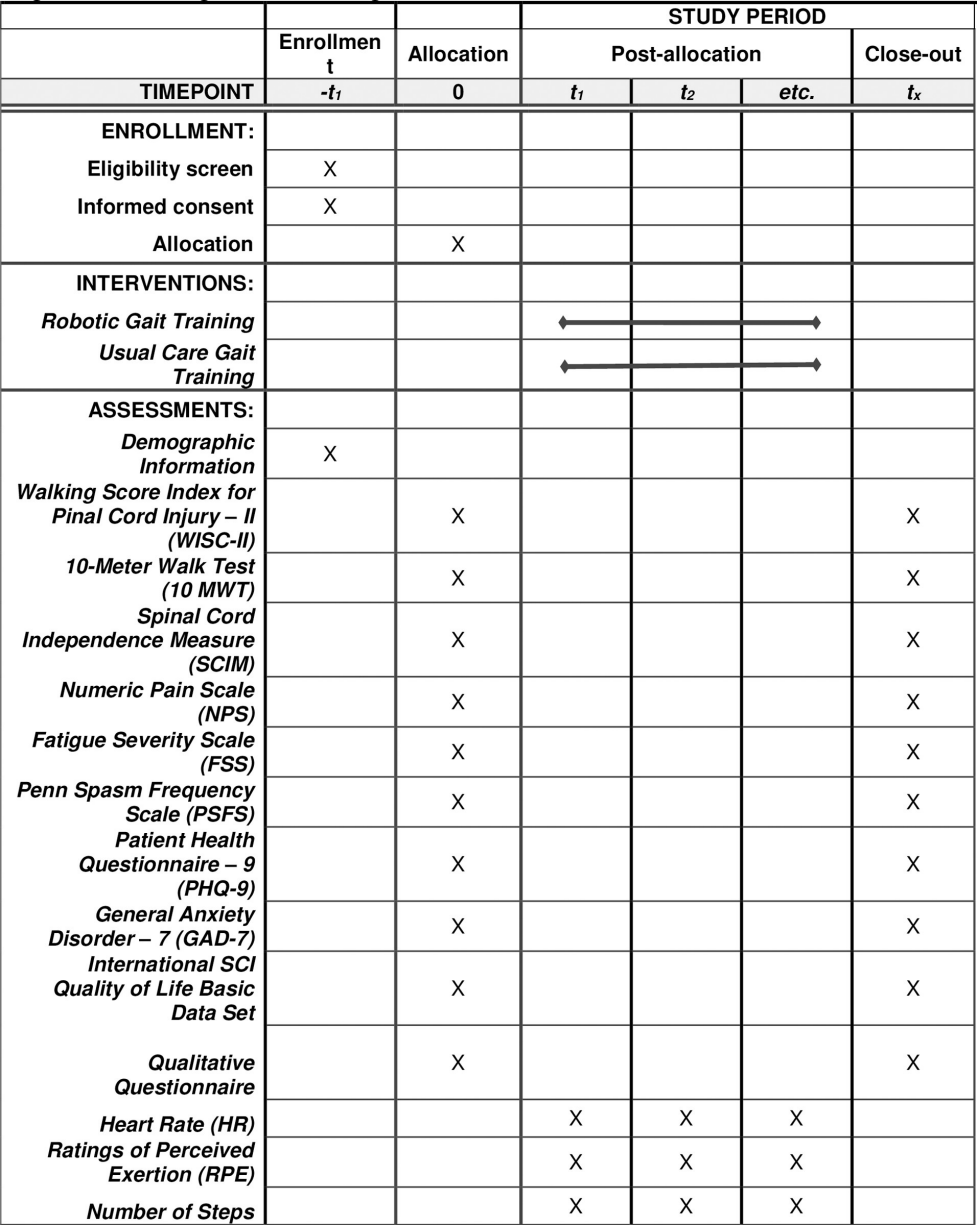
SPIRIT figure summarizing patient event timeline.

This is a prospective randomized controlled trial. Participants will be randomly assigned to one of two groups: RGT or UC. This study has been registered on ClinicalTrials.gov (Registration number: NCT04781621) and was approved by an Institutional Review Board (020–483). The intervention design and timeline are outlined in Fig 1.

### Study setting

This study will take place at a large rehabilitation hospital in an urban setting in the southwestern United States between April 2021 and August 2023. The rehabilitation hospital is accredited by The Joint Commission.

### Participants and recruitment

All patients with incomplete SCI will be screened at admission to inpatient rehabilitation at for initial eligibility.

Screening and patient suitability will be discussed at the twice weekly SCI clinical team rounding meeting. Study staff will approach identified individuals who meet the inclusion/exclusion criteria during the first 7 days in a private setting and briefly explain the study and answer any patient questions for clarification. Caregivers can consent to study on behalf of minors and minors will be given an opportunity to assent to project.

### Eligibility

Patients will be screened at admission for initial eligibility and qualifying patients will be approached to participate. Individuals between the ages of 16 to 85 years within six months post-incomplete SCI who are admitted to inpatient rehabilitation and who meet criteria to use a robotic exoskeleton will be recruited to participate in this study. Patients must meet manufacturer robotic exoskeleton frame limitations including weigh 100 kilograms or less; between 1.52 meters and 1.92 meters tall; have a standing hip width of 45.72 centimeters or less; have near normal range of motion in hips, knees, and ankles; able to attain a neutral ankle dorsiflexion with < 12 degrees of knee flexion; no more than 12 degree hip flexion contracture; no upper leg length discrepancy greater than 1.27 centimeters or lower leg discrepancy greater than 1.91 centimeters.

Exclusion criteria are as follows: (1) moderate to severe TBI, (2) degenerative diagnoses, (3) wound located in proximity to the exoskeleton frame, (4) severe osteoporosis/-penia as shown with DXA, (5) pre-morbid developmental disability, significant psychological diagnosis, or other cognitive impairment.

Patients will be evaluated by clinicians to determine if they meet the inclusion/exclusion criteria during their inpatient stay. If a patient is not initially appropriate for intervention due to medical reasons and later determined to appropriate during their stay, they may be approached to consent to participate.

### Intervention

Each patient with SCI enrolled in this study (both RGT and UC groups) will be provided with standard of care inpatient rehabilitation services. Patients who are admitted must be able to tolerate 3 hours of intense rehabilitation services per day [17]. During inpatient rehabilitation, and to meet CMS requirements [17], the dose for Inpatient Physical Therapy Services will be 90 minutes per day for 5 days per week of physical therapy, which is consistent with nationally recognized inpatient rehabilitation. The content of inpatient physical therapy services, individualized to participants’ needs based on level of function and medical need, may include basic mobility training (e.g., bed mobility, transfers), stretching and strengthening exercises, gait training (e.g., BWSTT, overground with braces), and wheelchair mobility. Therapeutic approaches follow both current SCI-specific clinical practice guidelines for rehabilitation and involve using only those approaches identified in a recent systematic review of 22 common therapeutic approaches for SCI as effective [18]. Refer to Table 1 for a sample schedule for physical therapy during inpatient rehabilitation.

**Table 1:** Sample schedule for physical therapy treatment: Usual care and RGT (in minutes).

Therapy Activity	Mon	Tues	Wed	Thurs	Fri	Sat	Sun	Total
Basic Mobility Training	30	30		30	30	30	30	180
Stretching and Strengthening Exercises		30	30			30	30	120
Gait Training								
Usual Care*	30		30		30			90
RGT**	30		30		30			90
Wheelchair Mobility		30	30	30				90
Electrical Modalities (e.g., FES)	30			30	30			90
Total Daily Physical Therapy	90	90	90	90	90	60	60	

*Gait training for the usual care (UC) group will consist of body weight–supported treadmill training and conventional overground walking for 90 minutes a week.

**Gait training for robotic gait training (RGT) group will include 90 minutes of RGT a week.

FES: functional electrical stimulation.

Both Treatment Groups receive equal dosage of recommended physical therapy approaches. Gait training for the UC group will consist of body weight–supported treadmill training (BWSTT) and conventional overground walking for 90 minutes a week. Gait training for RGT group will include 90 minutes of RGT a week. Each participant will engage in an intervention of gait training. Patients will participate for the duration of their length of stay for inpatient rehabilitation, from the time they consent after admission to the time they discharge.

### Outcome measures

Assessments will be conducted at admission, discharge and during the treatment intervention. Table 1 shows the patient event timeline for outcomes evaluated at different time points. The following demographic data is collected at admission: current age and age at injury; injury severity; gender; ethnicity; education level; pre-morbid history of mental illness; residence status; income; insurance type; vocation; financial status and ASIA Impairment Scale (AIS) score.

The primary and secondary outcome measures are listed in Table 2. The 10MWT, WISCI-II, SCIM, NPRS, and PSFS assessments will be assessed by a licensed clinician. Patients will complete the following self-reported assessments with a blinded research staff: FSS, PHQ-9, GAD-7, GAD-7 and Qualitative Questionnaire. After each treatment intervention, heart rate (HR) and number of steps will be collected from devices (Polar Watch, Pedometer, and EKSO) worn by patient and self-reported rating of perceived exertion (RPE).

**Table 2:** Outcome measures for proposed project and link to ICF.

**Primary Outcome**	
*Walking Index for Spinal Cord Injury–II (WISCI-II)	WISCI-II defines the physical limitation for gait secondary to impairment at the person level and indicates the ability of a person to walk after SCI [19]. Intrarater and interrater reliability are excellent at 1.0 and 0.98 respectively [20]. A change of one WISCI level can be considered clinically significant [21].
**Secondary Outcomes**	
**Gait speed via 10-Meter Walk Test (10MWT)**	Assesses gait speed over a short duration. Gait speed (m/s) is correlated ability to mobility in the community, capacity to perform activities of daily living, risk of falls, re-hospitalization, and risk of cognitive decline [22]. We will assess gait speed at inpatient rehabilitation admit and discharge, 3- and 6-month follow-up sessions to monitor longitudinal changes throughout the study. For persons with SCI, a combination of the 10MWT and the WISCI-II is recommended to provide the most valid measure of improvement in gait [23]. A change of >0.06 m/s is considered to exceed minimally clinically important difference (MCID) [24] and test-retest reliability is excellent (ICC = 0.97) [25].
**Spinal Cord Independence Measure (SCIM)**	The SCIM assesses self-care management, respiration and sphincter management, and functional mobility after a SCI. With excellent interrater reliability (r = 0.90) [26]. the SCIM is reported to be more sensitive to functional changes than the FIM [27].
**Numerical Pain Rating Scale (NPRS)**	Pain is a significant problem in many individuals with SCI. A 0–10 Point Numerical Pain Rating Scale (NRS) is recommended as the outcome measure for pain intensity after SCI during acute and subacute phases [28]. Pain severity can be categorized into 3 distinct groups as relates to pain interference: 1–3 (mild), 4–7 (moderate), 8–10 (severe) [29].
**Fatigue Severity Scale (FSS)**	The Fatigue Severity Scale (FSS) may be the most widely used measure of fatigue in neurologic disorders [30] and is a unidimensional measure that measures the effects of fatigue on function. The FSS is easy to use both in clinical practice and research, quick to administer, and its focus on the effects of fatigue on function makes its use in rehabilitation settings particularly appealing [31]. The FSS has acceptable reliability with regard to internal consistency, test-retest reliability, and validity in persons with SCI [31].
**Penn Spasm Frequency Scale (PSFS)**	The PSFS is a self-report measure to assess a patient’s perception of spasticity frequency and severity following a SCI. With excellent internal consistency (ICC = 0.90) [32], the current version was modified from the original to include both frequency and severity [33].
**Patient Health Questionnaire—9 (PHQ-9)**	The PHQ-9 is a self-report measure to assess the presence and intensity of depressive symptoms. For SCI, the PHQ-9 demonstrates excellent internal consistency (Chronbach’s alpha = 0.87) [34] and construct validity (r = 0.78) [35].
**General Anxiety Disorder– 7 (GAD– 7)**	The GAD-7 is a self-report measure to assess severity of anxiety symptoms over the past two weeks. When compared with SCI-QOL Anxiety, a correlation of 0.67 and reliability of 0.85 for the GAD-7 provides some support of its use after SCI [36].
**International SCI Quality of Life Basic Data Set**	The ISCIQOL Basic Data Set is a three-item quality of life questionnaire suitable for SCI populations containing 3 variables rating satisfaction with general quality of life, physical health, and psychological health [37]. Items are answered on a 10-point likert scale that ranges from 0 (completely dissatisfied), to 10 (completely satisfied).
**Qualitative Questionnaire**	Participants will be asked questions regarding their experience during the invention such as their likes and dislikes; notable changes or observations; and overall satisfaction with their care.
**Heart Rate (HR)**	Polar heart rate monitor (RS300X, Polar®) will provide data on the day, duration, and intensity (average and maximum) of gait training sessions for both RGT and UC. Participants will be provided a Polar heart rate monitor to wear during each gait training session for the entire length of the study, and gait training session data will be collected weekly. These monitors record beat to beat heart rates and store up to 16 sessions of heart rate data. Each week participants’ heart rate data will be uploaded using Polar’s FlowLink technology via the Polarpersonaltrainer.com website.
**Ratings of Perceived Exertion (RPE)**	The Borg RPE is a 15-point scale with verbal descriptors to standardize perceived exertion across tasks and individuals. Participants will be asked to provide a self-reported intensity level on the Borg Rating of Perceived Exertion Scale [38] during RGT and UC gait training sessions. A self-report of 12 to 14 on the RPE indicates moderate intensity. The Borg RPE scale has been shown to be a valid measure of exercise intensity with weighted mean validity coefficient of 0.62 for HR [39].
**Number of Steps**	The Ekso device records several data points for each session including number of steps, “Up” time (the amount of time spent standing in the device), “Walk” time (the amount of time spent walking in the device), and device assistance scores. While all of these data values will be recorded to describe each RGT session and tracked to monitor progression of the RGT intervention, the number of steps per session will be utilized as an indicator of RGT session intensity [40, 41]. Number of steps for the UC group will be collected via pedometer during each gait training session.

### Sample size

Sample size calculations were performed to determine the required sample size in each group based on detectable effect size. For 80% power with a 5% significance level, 64 participants will be enrolled in each group (n = 128 total) to detect a medium effect size of 0.5 and allow for ~10% attrition due to unplanned medical events, acute care transfers, and patient withdrawals. All analysis will be performed using SAS 9.4 significance set at the 5% level.

### Allocation

Participants will be randomized using cluster randomization based on the characteristic of level of injury (incomplete tetraplegia and incomplete paraplegia) for stratified block. Within each strata randomization blocks of size 6 will be used to ensure equal distribution between groups. The randomization scheme will be generated by the biostatistician, with the randomization scheme uploaded into the REDCap database by an unblinded data analyst.

### Data management, quality assurance, and exclusion of bias

Data management functions will occur on a quarterly basis and will include data quality checks and verification, as well as internal edit and logic checks (e.g., out of range values, internal inconsistencies). Ten percent of charts will be audited for source document and data entry review. Cross tabulation checks using SAS will also be used. Data will be stored and backed-up periodically on the biostatistician’s space on the secure server. Descriptive statistics will be at calculated and included into quarterly reports for a Data Safety and Monitoring Board to ensure the quality of data and progress of the study. In addition, a quality assurance coordinator independent of the study execution will conduct a quality assurance review at beginning of enrollment, midpoint and end of the study.

A detailed Data and Safety Monitoring Plan will be submitted to the IRB. The study staff will be responsible for collecting and recording all clinical data. As results are collected, all Adverse Events will be identified, graded for severity and assigned causality, reported to the required entities, and compiled for periodic review. To protect confidentiality, all subjects will be given unique subject IDs which will be used for all study documentation. All dates will be calculated and identifiers will be deleted. Links to identifiers will be kept on a secure server in a separate password-protected file and will only be available to study personnel. All study data will be input into REDCap database which is a secure, HIPAA-compliant web application to manage research data. No identifying information will be used for statistical analyses. All scientific data will be submitted to ICPSR per ACL/NIDILRR guidelines following the completion of the study.

Given the type of intervention, participants will not be blinded to group assignment. However, to minimize assessor bias, outcome assessments will be conducted by blinded research staff.

### Statistical methods

**Aim 1**: Develop an RGT program that meets the unique needs of people after incomplete SCI during inpatient rehabilitation.

**Aim 1 Analysis.** Engagement of the Advisory Board in development of the RGT program and recommendations throughout the 3-year study will be qualitatively described.

**Aim 2**: Examine the efficacy of RGT compared to UC gait training during inpatient rehabilitation in people with incomplete SCI.

**Aim 2 Analysis.** All analysis will be performed using SAS 9.4 with a significance level of 0.05. To determine if patients with incomplete SCI who receive RGT will have higher walking function than UC at discharge (Hypothesis 2.1), WISCI-II scores will be evaluated using a linear model. The distribution of WISCI-II scores will be assessed to determine if a general linear model will be utilized, or if a generalized linear model with an alternative distribution and link function, such as the gamma distribution with a log link, will be more appropriate. Similarly, all measures associated with the secondary outcomes in Hypothesis 2.2 (10MWT, SCIM, NPRS, FSS, PSFS, PHQ-9, GAD-7, and International SCI Quality of Life Basic Data Set Qualitative Questionnaire) will be evaluated with general or generalized linear models, as appropriate. A separate model will be run for each outcome. All models for both the primary and secondary outcomes will control for scores at admission as well as the patient’s demographic and impairment information listed in section B.1.i.D.

**Aim 3**: Compare the intensity of RGT and UC gait training during inpatient rehabilitation. Intensity data will include (1) heart rate, (2) RPE, and (3) number of steps.

**Aim 3 Analysis:** Moderate heart rate intensity minutes, average RPE, and steps will be evaluated using individual general or generalized linear models, as appropriate, to determine the association between RGT and UC and intensity. Each model will control for the patient characteristics and total number of sessions. Additionally, to determine the relationship between session intensity and walking function correlation coefficients will be calculated between each intensity measure and WISCI-II at admission, discharge, and change score.

For both Aims 2 and 3 stratified subset analysis will be performed for tetraplegia and paraplegia to determine if RGT has a differing impact depending on injury classification.

If a patient misses an assessment, their data for that outcome will be treated as missing and will not be included in the statistical model. However, the data from the completed assessments for that patient will be included. Sensitivity analysis will be performed to assess the impact of missing data, by imputing a missing outcome measure using the overall average change score for the given outcome. The sensitivity analyses will be compared to the initial analysis to determine the impact of missingness on the results.

## Discussion

The ability to walk is a priority for people with SCI [42] particularly among those newly injured. While there is great interest in the potential of pharmacological, biological, and genetic interventions to improve walking function in persons with SCI [43], the efficacy of these approaches has not yet been established. A recent systematic review of strategies to improve motor function in persons with SCI concluded that gait training is a necessary component for recovering walking function in multi-intervention approaches [43]. However, the most effective gait training approach for people with SCI remains unclear. The overarching goal of this protocol is to address walking recovery among those with SCI during inpatient rehabilitation by developing and evaluating an inpatient rehabilitation RGT program for people with SCI when the neuroplasticity potential is greatest [44]. The study offers a foundational step in establishing a stakeholder-informed clinical protocol for RGT use in this setting and evidence of the effectiveness of the approach compared to usual care. This study will yield evidence about the clinical use and effectiveness of a stakeholder informed RGT program during inpatient rehabilitation for people with incomplete SCI. Study findings will be disseminated through patient and clinician centered fact sheets, scientific conferences, and peer-reviewed publications.

## Supporting information

S1 Checklist SPIRIT checklist. (DOC)

S1 Protocol FIRST protocol V3. (DOCX)
