# Supplementary material for: Foundational ingredients of robotic gait training for people with incomplete spinal cord injury during inpatient rehabilitation (FIRST): A randomized controlled trial protocol
Source: PLoS One. 2022 May 10;17(5):e0267013. doi: 10.1371/journal.pone.0267013 (PMC9089894; doi:10.1371/journal.pone.0267013)
Supplement: S1 Protocol — (DOCX) [file pone.0267013.s002.docx]

| **Protocol Title** | **F**oundational **I**ngredients of **R**obotic Gait Training for People with **S**pinal Cord Injury During Inpatient **T**herapy (FIRST) |
| --- | --- |
| **Version Number** | V3.0 |
| **Date** | 6/4/2021 |
| **IRB Number** | 020-483 |
| **National Clinical Trials #** | NCT04781621 |
| **Sample Size** | 128 |
| **Sponsor** | National Institute on Disability, Independent Living, and Rehabilitation Research (NIDILRR) |
| **Principal Investigator** | Chad Swank, PhD, PT, NCS |
| **Study Coordinator** | Alexandria Holden |

**Table of Contents**

[I PROTOCOL SYNOPSIS 3](#_Toc57819594)

[1 LIST OF ABBREVIATIONS & DEFINITIONS OF TERMS 3](#_Toc57819595)

[2 BACKGROUND AND RATIONALE 3](#_Toc57819596)

[3 STUDY AIMS 6](#_Toc57819597)

[4 INVESTIGATIONAL PLAN 6](#_Toc57819598)

[5 STUDY PROCEDURES 12](#_Toc57819599)

[6 STUDY MEASUREMENTS 15](#_Toc57819600)

[7 STUDY TERMINATION 17](#_Toc57819601)

[8 DATA QUALITY ASSURANCE 17](#_Toc57819602)

[9 STATISTICAL METHODS 18](#_Toc57819603)

[11 INVESTIGATOR’S RESPONSIBILITIES 19](#_Toc57819604)

[12 ETHICS 20](#_Toc57819605)

[13 STUDY DOCUMENTATION AND RECORDKEEPING 21](#_Toc57819606)

[14 USE OF INFORMATION AND PUBLICATION 23](#_Toc57819607)

[15 REFERENCES 24](#_Toc57819608)

# **I PROTOCOL SYNOPSIS**

| **Site Project Summary: F**oundational **I**ngredients of **R**obotic Gait Training for People with **S**pinal Cord Injury During Inpatient **T**herapy (FIRST) |
| --- |
| **Importance:** Spinal cord injury (SCI) due to trauma is estimated to affect 288,000 – 500,000 Americans, with about 17,700 new cases annually. Despite general advances in medicine, the average life expectancy for persons with SCI has remained largely unchanged since the 1980s and are significantly lower than for persons without SCI. Given this disparity of life expectancy and decreased quality of life after SCI, gait training has the opportunity to positively impact the lives of people after SCI. Recent technological advances with exoskeleton devices specifically for gait training may yield better walking recovery outcomes compared with current intervention approaches, such as body-weight support treadmill training (BWSTT) and overground gait training with braces, yet limited evidence exists for those with SCI. |
| **Aims:** (1): Use a Community-Based Participatory Research approach to develop a Robotic Gait Training (RGT) program that meets the unique needs of people after incomplete SCI during inpatient rehabilitation. (2): Prospectively examine the efficacy of RGT compared to usual care gait training (UC) during inpatient rehabilitation in people with incomplete SCI. (3): Compare the intensity of RGT and UC gait training during inpatient rehabilitation. |
| **Method:** Patients will be randomized to either the experimental group using Robotic Gait Training (RGT) or the control group receiving usual care (UC). We will enroll 128 patients admitted to Baylor Scott & White Institute of Rehabilitation for inpatient rehabilitation services. |
| **Addition to State-of-the-Art:** Successful completion of this study will provide an evidence-based intervention, specifically tailored to meet the unique needs of people with SCI, which supports walking recovery; maximizing health, function, and ultimately participation. The intervention will further support widespread clinical implementation of exoskeleton use during acute rehabilitation. |
| **Sustained Approach:** This study builds on our prior experience of gaiting training and therapeutic recommendations for individuals with SCI over the past several years. If successful, we plan to incorporate the best gait training approach with the most robust outcomes while also being concerned with patient tolerance and clinical outcomes. |

# **1 LIST OF ABBREVIATIONS & DEFINITIONS OF TERMS**

SCI – Spinal Cord Injury

BSWIR- Baylor Scott & White Institute for Rehabilitation

# **2 BACKGROUND AND RATIONALE**

**Background:** Dramatic advances in technology are being adopted into clinical rehabilitation practice at a rapid pace. Robotic exoskeletons (wearable robots to enhance overground mobility) are a potentially viable technology that can be used for gait training among those with acute spinal cord injury (SCI). Yet, foundational evidence to support exoskeleton use during the acute phase of recovery is lacking . While robotic exoskeleton technology is not yet mature enough to produce independent community ambulation,^1,2^ the technology has improved^3^ and may allow for enhanced SCI rehabilitation.^4^ Over the past 20 years robotic exoskeleton use has primarily been studied in people living with chronic SCI, with early evidence demonstrating that use improves gait, bone density, body composition, and specific cardiovascular parameters.^5^ One exciting, yet understudied, application of robotic exoskeletons in rehabilitation is the possibility of enhancing functional outcome by using the device during the acute recovery phase,^6^ when the central nervous system’s ability to reorganize itself by forming new neural connections, known as neuroplasticity, is greatest and changes in functional recovery most likely.^7^ Notably, more than half of expected recovery occurs in the first two months after SCI and subsequent improvement plateaus after 3 to 6 months.^8^ This critical period of neuroplasticity suggests that functional recovery may be greater using rehabilitation approaches that maximize the potential for generating neuroplasticity. Initial evidence of robotic exoskeleton use that our team at Baylor Scott & White Institute for Rehabilitation (BSWIR) has generated demonstrates that this technology may offer a better approach for gait training that yields improved functional outcomes over traditional gait training approaches. This study proposed in this application systematically builds upon our initial evidence examining the feasibility and effect of robotic gait training (RGT) during acute rehabilitation to improve walking recovery after SCI.

Our initial findings offer intriguing evidence suggesting that RGT can (1) be feasibly integrated into clinical practice during inpatient rehabilitation without adverse effects,^9^ (2) yield greater improvements in gait than usual care measured by changes in the Functional Independence Measure (FIM) and the Walking Index for Spinal Cord Injury-Revised (WISCI-II),^10,11^ (3) elicit more time spent in moderate intensity exercise based on heart rate reserve (~66%) when compared to usual care gait training (~42%), functional exercises (~27%), stationary cycling (~18%), and strength training (~5%)^12^ and (4) promote gait patterns with reduced asymmetry and appropriate electromyography (EMG) activity.^13,14^ While our initial data are promising, the evidence base is insufficient to support the contention that RGT can yield significantly better outcomes than traditional rehabilitation approaches during the acute stage of recovery. This is particularly salient due to the opportunity to harness the body’s potential for neuroplasticity during inpatient rehabilitation. Our proposed study will address this gap in science by providing data on the active components of a RGT program developed with input from an Advisory Board to meet the unique needs of people with SCI undergoing inpatient rehabilitation.

**Functional gait deficits faced by people with incomplete spinal cord injury (SCI):** Spinal cord injury (SCI) due to trauma is estimated to affect 288,000 – 500,000 Americans, with about 17,700 new cases annually.^15^ Despite general advances in medicine, the average life expectancy for persons with SCI has remained largely unchanged since the 1980s and are significantly lower than for persons without SCI.^16,17^ Given this disparity of life expectancy and decreased quality of life after SCI,^18^ gait training has the opportunity to positively impact the lives of people after SCI. Evidence demonstrates that gait training results in significant functional improvements for those with traumatic and non-traumatic SCI, but the degree of functional improvements are dependent on the nature of the non-traumatic etiology.^19^ Nevertheless, despite the cause of injury to the spinal cord, the effects impact transmission of impulses along the motor and sensory pathways and typically results in substantial mobility impairment including walking.^20^ Not surprisingly, recovery of walking is a primary rehabilitation goal for patients and encouraged by therapists^21,22^ due to its relationship to quality of life,^23,24^ impact on health, psychological profile,^25^ and social participation after SCI.^26,27^. Recent technological advances with exoskeleton devices specifically for gait training may yield better walking recovery outcomes compared with current intervention approaches, such as body-weight support treadmill training (BWSTT) and overground gait training with braces, yet limited evidence exists for those with SCI.

**Need for evidence-based gait training interventions that specifically address the unique needs of people with incomplete SCI during inpatient rehabilitation:** The ability to walk is a priority for people with SCI ^28^ particularly among those newly injured. While there is great interest in the potential of pharmacological, biological, and genetic interventions to improve walking function in persons with SCI,^29^ the efficacy of these approaches in humans has not yet been established. A recent systematic review of strategies to improve motor function in persons with SCI concluded that **gait training is a necessary component for recovering walking function in multi-intervention approaches.^29^ However, the most effective gait training approach for people with SCI remains unclear.** Recent systematic reviews demonstrate equivocal outcomes between approaches to walking recovery including BWSTT, robotic assisted gait training (i.e. Lokomat), and manually assisted overground gait training for people with sub-acute SCI^30^ or chronic SCI.^31,32^ Additionally, usual care gait training interventions (e.g., BWSTT, manually assisted overground) lack a strong evidence base for people with incomplete SCI despite the widespread use.^33^

Emerging evidence suggests there are **several critical ingredients** to gait training specific to people with SCI. **First**, volitional effort of the participant is essential. Robotic assisted training wherein full assistance is provided (e.g., Lokomat) and stepping is produced without volitional effort from the participant is less effective than methods requiring volitional effort (e.g., RGT).^34,35^ **Second**, interventions that are intended to improve walking function are most effective when they incorporate task-specific gait training. Thus, directly engaging the neural circuits required for walking has a greater impact on walking speed.^36^ **Third**, gait training intensity is vital because it improves cardiometabolic health^37^ and promotes neuroplasticity,^38-41^ and the recommended approach for tracking intensity is recording the number of steps during gait training sessions.^42^ **Unlike usual care gait training approaches, robotic exoskeleton technology integrates these three critical ingredients of gait training after SCI**. Specifically, robotic exoskeletons promote volitional effort through variable assistance features^44^, encourage task-specific gait training,^45^ and require moderate exercise intensity across sitting, standing, and walking tasks.^46^ Despite established principles of neuroplasticity,^47,48^ and that these critical ingredients to gait training are integrated into robotic exoskeleton use, clinical recommendations to support the use of RGT during inpatient rehabilitation are lacking. This clinical gap is largely due to the lack of foundational evidence around RGT use in acute rehabilitation, emphasizing the need for research in this area. **Thus, the overarching goal of this study is to address the current gap in the literature by generating and testing a RGT program to improve walking recovery during inpatient rehabilitation.**

**Rationale for Protocol:** The overarching goal of this study is to improve walking recovery among those with SCI during inpatient rehabilitation by developing and evaluating an inpatient rehabilitation RGT program for people with SCI when the neuroplasticity potential is greatest.^60^ The current study offers a foundational step in establishing a clinical protocol for RGT use in this setting and evidence of the effectiveness of the approach compared to usual care. Our proposed approach to develop and examine clinical recommendations for RGT during inpatient rehabilitation will be informed by evidence from our initial work in this area, current clinical practice, and stakeholder guidance. As those with first-hand knowledge and experience of SCI are critical sources of information describing and contributing to program development,^61^ we seek to fill the evidence gap by including stakeholder perspectives through an Advisory Board that will be engaged with the project across all funding years.

We anticipate the **short-term impact** to (1) improve gait outcomes for people with incomplete SCI during inpatient rehabilitation (Aims 1 and 2) and (2) describe the intensity of our RGT intervention for people with incomplete SCI during inpatient rehabilitation (Aim 3). We anticipate the **long-term impact** to (1) generate data for informing and developing training protocols to improve walking function after SCI, and (2) provide foundational data to evaluate the dose-response relationship of RGT in the inpatient setting. The successful completion of this project will yield evidence about the clinical use and effectiveness of RGT during inpatient rehabilitation for people with incomplete SCI.

# **3 STUDY AIMS**

**Aim 1:** Use a Community-Based Participatory Research approach to develop an RGT program that meets the unique needs of people after incomplete SCI during inpatient rehabilitation. This will be achieved by establishing and engaging an Advisory Board of key stakeholders (patients, caregivers, clinicians, researchers, industry members) to review evidence-based literature, advise the research team on the unique aspects and goals of inpatient rehabilitation for people with SCI, review the RGT, and make recommendations for amendments to the RGT program based on our interim and final outcomes of the study over the funding period.

Hypothesis 1.1: *We will successfully engage key stakeholders in a community-based participatory research approach to develop the content and structure of the RGT program to accommodate current clinical practice guidelines and integrate emerging robotic exoskeleton technology.* Our hypothesis is based on the fact we have previously demonstrated the successful inclusion of RGT into clinical practice during inpatient rehabilitation and detected change in key outcome variables.

**Aim 2**: Prospectively examine the efficacy of RGT compared to usual care gait training (UC) during inpatient rehabilitation in people with incomplete SCI.

Hypothesis 2.1: *Patients with incomplete SCI who receive RGT will have higher walking function [Walking Index for Spinal Cord Injury – Revised (WISCI-II)] at discharge compared to UC.* Our hypothesis is based on our previous work showing SCI patients who received 5+ RGT sessions demonstrated greater improvement on the WISCI-II compared to UC.^10,11^

Hypothesis 2.2*: Patients with incomplete SCI who receive RGT will have greater improvement in secondary outcomes (gait speed, daily functional independence, pain, fatigue, spasm frequency, depressive symptoms, and quality of life) at discharge compared to UC.*

**Aim 3**: Compare the intensity of RGT and UC gait training during inpatient rehabilitation. Intensity data will include (1) heart rate, (2) rate of perceived exertion (RPE), and (3) number of steps.

Hypothesis 3.1: *RGT will be of significantly greater intensity with participants (1) spending more minutes per session and total minutes (over all sessions) in moderate intensity exercise (40% to 60% range of heart rate reserve), (2) reporting higher mean RPE across all sessions, and (3) completing more steps during each session and total number of steps over all sessions compared to UC.* Our hypothesis is based on the theoretical rationale described in **Tables 3** and **Table 4** below, our previous work,^12^ and will incorporate input from the Advisory Board regarding these variables and adjust the intensity as suitable.

Hypothesis 3.2: *Intensity will be significantly correlated to the primary outcome (WISCI-II) for the RGT and UC groups.*

# **4 INVESTIGATIONAL PLAN**

**Study Design**

Randomized Controlled Trial (RCT)

**Study Treatment**

Each patient with SCI enrolled in this study (both RGT and UC groups) will be provided with Inpatient Rehabilitation Services as is standard practice at BSW. Patients who are admitted must be able to tolerate 3 hours of intense rehabilitation services per day.^92^ During inpatient rehabilitation, and to meet CMS requirements^92^, the dose for Inpatient Physical Therapy Services will be 90 minutes/day for 5 days/week of physical therapy, which is consistent with nationally recognized inpatient rehabilitation. The content of Inpatient Physical Therapy Services, individualized to participants’ needs based on level of function and medical need, may include basic mobility training (i.e. bed mobility, transfers), stretching and strengthening exercises, gait training (e.g., BWSTT, overground with braces), and wheelchair mobility. These are consistent with evidence-based approaches for people with SCI. BSW PT therapeutic approaches follow both current SCI-specific clinical practice guidelines for rehabilitation and involve using only those approaches identified in a recent systematic review of 22 common therapeutic approaches for SCI as effective.^33^ Thus, the content of Inpatient Physical Therapy Services for our patients with SCI will be consistent with these recommendations, as is standard practice at BSW. Each participant will also be randomized into one of two groups for gait training approach: (1) Usual Care (UC) and (2) Robotic Gait Training (RGT).

Both Treatment Groups receive equal dosage of recommended physical therapy approaches. Gait training for the Usual Care (UC) group will consist of body weight–supported treadmill training (BWSTT) and conventional overground walking for 90 minutes a week. Gait training for robotic gait training (RGT) group will include 90 minutes of RGT a week. Each participant will engage in an intervention of gait training.

*UC*

UC gait training at BSWIR adheres to the current SCI-specific clinical practice rehabilitation guidelines. These guidelines recommend that body weight–supported treadmill training (BWSTT) as an option for ambulation training in addition to conventional overground walking, dependent on resource availability, context, and local expertise.^65^ BSWIR currently offers 2 BSWTT systems, 1 overground body weight-supported overground system, and various assistive devices (e.g., walkers, leg braces) to perform manually assisted overground gait training. BSW therapists are trained and proficient in providing UC gait training interventions to patients with SCI. Patients in the UC group will receive 90 minutes per week of UC gait training (e.g., BWSTT, overground with braces).

If participants are randomized into the usual care group and have completed care, they will be offered up to eight optional sessions of robotic walking training in outpatient therapy at BSWIR in Dallas, Texas. Participants will need to obtain a physician referral and arrange their own transportation.

*RGT*

Patients in the RGT group will receive 90 minutes per week of RGT in the Ekso device once patients are deemed clinically appropriate as defined by being able to tolerate standing for 15 minutes without orthostatic intolerance. The Ekso Bionics Ekso GT™ robotic exoskeleton will be used for RGT.

The EksoGT™ robotic exoskeleton is a class II medical device (United States FDA) that allows patients with SCI to experience over-ground weight bearing stepping.^94^ The Ekso is a battery powered wearable bionic suit with motors at the hips and knees which enables individuals with lower extremity motor impairment to stand and voluntarily step over-ground with weight-bearing and alternating stepping (see Figure 2). The Ekso device works in two modes namely the “max assist” and “variable assist.” In max assist, the pilot (patient) initiates a step by unweighting one leg which triggers the motors on the Ekso to move the entire limb in a step like trajectory. In this mode, 100% assistance is offered during the entire step cycle. The variable assist mode actively allows the subject to voluntarily assist, even when the subject exerts minimal voluntary influence on the robot. While stepping with the variable assistance mode, once the subject initiates a step, the Ekso only provides assistance during the swing phase based on the amount of effort the patient applies. An essential advantage of overground robotic exoskeleton devices (e.g., Ekso, Indego) over treadmill-based exoskeletons (e.g., Lokomat) is the ability to voluntarily engage the lower limbs while stepping, by engaging both supraspinal and spinal networks in a synergistic manner (*critical ingredient #1, pg 2*). This “assist as needed” paradigm allows the subject to engage the lumbosacral spinal networks to control one limb at a time to complete a swing phase while the contralateral limb goes through a passive stance phase.^94^ In the proposed study, the variable assist mode will be used exclusively. We elected to use the Ekso exclusively in this ‘intervention development’ study because the FDA indications for use accommodate a broader range of injury levels of SCI compared to other FDA approved overground robotic exoskeleton devices.

During the first RGT session, the patient will be fitted for the Ekso device. RGT will be initiated immediately thereafter. While each training session may vary in length due to medical acuity and patient tolerance, RGT will total 90 minutes/week. Based on our retrospective data, we anticipate the specific tasks during RGT to vary between standing tasks and walking, with more steps and time spent walking as the RGT sessions progress. Notably, the RGT records data for each training session including frequency, time standing, time walking, and number of steps.

Progression of the RGT intervention will be individualized to accommodate injury severity characteristics and variable rates of functional recovery. However, a general approach to progression of RGT will include the following sequential steps: (1) familiarization with Ekso stepping and weight shift pattern, (2) standing tasks > walking tasks, (3) walking tasks > standing tasks, (4) program reduced maximal assistance provided by Ekso during walking, (5) program zero assistance provided by Ekso during walking, (6) program resistance provided by Ekso during walking. This progression is based on recommendations during Ekso training and our therapists’ clinical experience.^81,82^

All RGT activities will be conducted with the necessary supervision and assistance from a licensed physical therapist trained in the use of Ekso robotic exoskeleton.

BSW owns 2 Ekso robotic exoskeleton devices for research purposes that will be used in this study. Extended service contracts will be purchased to ensure the continual maintenance of the Ekso devices for the duration of the study. Ten physical therapists have been trained to use the Ekso for mobility and gait training, successfully completing Level I (basic) and Level II (advanced) training under the supervision of an Ekso trainer. The therapists have an average of nearly 2-years’ experience conducting RGT and 7.5 years of inpatient rehabilitation experience working with patients with SCI.

**Table 1. Sample Schedule for Physical Therapy Treatment: Usual Care and RGT (in minutes)**

| **Therapy Activity** | **Monday** | **Tuesday** | **Wednesday** | **Thursday** | **Friday** | **Saturday** | **Sunday** | **Total** |
| --- | --- | --- | --- | --- | --- | --- | --- | --- |
| Basic Mobility Training | 30 | 30 |  | 30 | 30 | 30 | 30 | 180 |
| Stretching And Strengthening Exercises |  | 30 | 30 |  |  | 30 | 30 | 120 |
| Gait Training |  |  |  |  |  |  |  |  |
| Usual Care* | 30 |  | 30 |  | 30 |  |  | 90 |
| RGT** | 30 |  | 30 |  | 30 |  |  | 90 |
| Wheelchair Mobility |  | 30 | 30 | 30 |  |  |  | 90 |
| Electrical Modalities (TENS, FES) | 30 |  |  | 30 | 30 |  |  | 90 |
| Total Daily Physical Therapy | 90 | 90 | 90 | 90 | 90 | 60 | 60 |  |

Note: ^1^ Both Treatment Groups receive equal dosage of recommended physical therapy approaches. *Gait training for the Usual Care (UC) group will consist of body weight–supported treadmill training (BWSTT) and conventional overground walking for 90 minutes a week. **Gait training for robotic gait training (RGT) group will include 90 minutes of RGT a week.

**Selection of Study Population**

**Recruitment**

All patients with incomplete SCI will be screened at admission to inpatient rehabilitation for initial eligibility. Typical inpatient rehabilitation length of stay at BSWIR varies based on factors including what is deemed medically appropriate by the SCI Medical Director (Dr. Sikka, Co-Investigator), approval by the insurance company, medical complexity, and rate of recovery. However, the average length of stay over the past year at BSW was 35 days for traumatic SCI and 22 days for non-traumatic SCI. Usual care rehabilitation is initiated within 24 hours of admission. Standard research recruitment procedures for our program may include family education materials and research flyers, as well as research coordinators educating clinicians and patients/families. Dr. Sikka will support recruitment through team conference and patient rounding.

**Subject Information and Screening** - We will screen all patients with SCI upon admission for initial eligibility (see **Table 2** and **3** for inclusion & exclusion criteria). Screening and patient suitability will be discussed at the twice weekly SCI clinical team rounding meeting. Study staff will approach identified individuals who meet the inclusion/exclusion criteria during the first 7 days in a private setting and briefly explain the study and answer any patient questions for clarification. The patient will be given adequate time to determine if they want to participate in the study.

**Study Period**

Patients will participate for the duration of their length of stay for inpatient rehabilitation, from the time they consent after admission to the time they discharge.

**Rationale for Selection of Dose**

Our proposed project is also rooted in principles of neuroplasticity ^(47, 48)^ (**Table 2**) and exercise prescription dose^(88)^ (**Table 3**). The 10 principles of neuroplasticity and 4 principles of exercise prescription dose – **especially specificity, intensity, and timing** – are driving forces to initiate RGT during inpatient rehabilitation. With clear resonance between neuroplasticity and exercise dose principles, this project will describe and relate RGT dose parameters with gait and functional outcomes.

| **Table 2: Link between Principles of Neuroplasticity and specific aims** | | |
| --- | --- | --- |
| **Principle** | **How Principle is Addressed in the Robotic Gait Training (RGT) Program** | **Specific Aim** |
| *Use it or lose it* | Initiation of RGT as integrated into a comprehensive rehabilitation program when patients meet inclusion criteria. Intent to stimulate specific brain and spinal cord functions with task specific activities as soon as possible to prevent CNS pathway degradation. | AIM 2 |
| *Use it and improve it* | RGT facilitates the patient for task specific gait activity over the extended time of the inpatient rehabilitation stay. | AIM 2 |
| *Specificity* | RGT specifically address the individual patient’s mobility and movement patterns to aid reach the patient reach his/her goals. | AIM 2 |
| *Repetition matters* | RGT activities are completed in addition to skilled therapy intervention while in the inpatient rehabilitation setting. The patient repeats the RGT over several sessions, therefore developing greater patterns for recovery during inpatient rehabilitation stay and upon discharge. | AIMS  2 & 3 |
| *Intensity matters* | RGT is implemented based upon individual patient activity tolerance. RGT intensity (e.g., heart rate, # of steps, up time, walk time) is assessed daily and modified by therapist. Patients are verbally encouraged to continue increasing intensity as tolerated by staff. | AIMS  2 & 3 |
| *Time matters* | The program is initiated for appropriate patients during inpatient rehabilitation when the potential for neuroplasticity is greatest.^60^ | AIMS  2 & 3 |
| *Salience matters* | RGT is a gait task specific activity and integrates into the patient’s function and goals. Patients are encouraged by continued awareness of successful activity either by therapist, family, or another patient involved in program. | AIMS  1 & 2 |
| *Age matters* | Patients begin station activities as soon they meet inclusion criteria and activities are specifically selected for the individual patient’s need. |  |
| *Transference* | Based on clinical practice guidelines, robotic exoskeleton literature, and our prior work, the physical therapist facilitates the RGT program activities for the patient that are strategically chosen to allow for integration and progression from robotic gait training to overground gait training. | AIMS  2 & 3 |
| *Interference* | The RGT program focuses on specific motor recruitment, movement patterns, and maintenance of range of motion to decrease maladaptive plasticity and disruptions of learning. | AIM 2 |

| **Table 3. Link between Principles of Exercise Prescription and specific aims** | | |
| --- | --- | --- |
| **Principle** | **How Principle is Address in the Robotic Gait Training (RGT) Program** | **Specific Aim** |
| *Frequency (F)* | The anticipated frequency of the RGT sessions will be 3x/week. This is based on American College of Sports Medicine cardiorespiratory guidelines for people with SCI^88^ and preliminary results from our Feasibility and Retrospective studies. | AIMS  2 & 3 |
| *Intensity (I)* | We will monitor and assess intensity of RGT sessions using the following methods:^88,89^ heart rate (percent of maximal HR), Ratings of perceived exertion (RPE),^90^ and step count.^42,91^ For cardiometabolic health benefits, people with SCI are recommended to engage in moderate to vigorous intensity of aerobic exercise.^88^ As a rule of thumb, a person doing moderate-intensity aerobic exercise can talk, but not sing, during the activity. A person doing vigorous-intensity exercise cannot say more than a few words without pausing for a breath. | AIMS  2 & 3 |
| *Time (T)* | The anticipated duration of the RGT sessions based on preliminary results from our Feasibility and Retrospective studies will be up to 45 min/session. | AIMS  2 & 3 |
| *Type (T)* | The mode of exercise will be walking in the Ekso during RGT. Interventions that are intended to improve walking function are most effective when they incorporate task-specific gait training to directly engaging the neural circuits required for.^36^ | AIMS  2 & 3 |

**Eligibility Criteria**

Individuals (n = 128) between the ages of 16 – 85 years within 6 months post-incomplete SCI who are admitted to BSW and who meet criteria to use an EKSO robotic exoskeleton will be recruited to participate in this study. Subjects must meet Ekso robotic exoskeleton frame limitations unless per the discretion of the PI it is determined that subject’s weight and/or height being outside of this range does not interfere with the fit of the device. Patients will be screened at admission for initial eligibility and qualifying patients will be approached to participate. Patients will be evaluated by clinicians to determine if they meet the inclusion/exclusion criteria during their inpatient stay. If a patient is not initially appropriate for intervention due to medical reasons and later determined to appropriate during their stay, they may be approached to consent to participate. In a private setting, patients, and their caregivers if applicable, will be approached about the study and a study team member will briefly explain the study and answer any patient questions for clarification. Caregivers of minors will sign consent form and minors will be given an opportunity to assent to project. Inclusion/exclusion listed below.

| **Table 4. Eligibility Criteria** | |
| --- | --- |
| **Inclusion Criteria** | **Rationale** |
| 16-85 years of age | Broad range for generalizability; Our inpatient rehabilitation facility admits patients with SCI who are 16 years and older. Patients who are 16 and 17 years of age are likely to receive similar gait interventions as adults aged 18+ and able to meet manufacturer height and weight specifications of the Ekso robotic exoskeleton device. This is consistent with previously published exoskeleton research.^1^ 97% of patients at BSW were between 18-70 with average age of SCI admissions over the past 5 years being 56.0 ± 18.6 years. |
| All types of incomplete SCI (traumatic and non-traumatic) | Broad range for generalizability. In this study, we will include people with incomplete SCI regardless of presence of tetraplegia or paraplegia (~68% of all people with SCI)^15^ because of the greater potential for motor recovery. |
| Acute/Subacute phase of recovery | We will include patients with incomplete SCI admitted to BSW for inpatient rehabilitation within 6 months post-SCI (~88% of admissions over the past 5 years). |
| Medically stable as deemed by physician | Cardiac risk factors will be considered by the Medical Director of the SCI Team as well as additional co-morbidities |
| Undergoing medical care and rehabilitation at BSW | Patients with SCI will be accessible for recruitment. 1,128 patients with SCI were admitted for rehabilitation at BSW over the past 5 years. |
| Both genders and all races and ethnicities | Achieve a representative sample based on the population admitted at BSW (64% white, 36% minority past 3 years) and national statistics (78% of new cases of SCI are male).^15^ |
| Meet the Ekso robotic exoskeleton frame limitations | Frame limitations include: weigh 220 pounds (100 kilograms) or less; between 5’0” and 6’4” tall; have a standing hip width of 18” or less; have near normal range of motion in hips, knees, and ankles; able to attain a neutral ankle dorsiflexion with < 12 degrees of knee flexion); no more than 12 degree hip flexion contracture; no upper leg length discrepancy greater than half an inch (> .5”) or lower leg discrepancy greater than three-quarters of an inch (>.75”). |
| Continence of or a program for bladder and bowel management | Incontinence may contaminate the robotic exoskeleton and prevent its continued use in the study. While the soft padding may be able to be cleaned, it is possible that body fluids associated with incontinence may ruin the mechanical joints and actuators. All of our patients with SCI are either continent or on a bladder and bowel program. |

**Exclusion Criteria**

| **Table 5: Exclusion Criteria** | |
| --- | --- |
| **Exclusion Criteria** | **Rationale** |
| Moderate to severe TBI | Issues with central executive functions which can affect an individual’s memory for new information, planning, task switching, and action monitoring. |
| Degenerative diagnoses | Functional improvements are dependent on the nature of the non-traumatic etiology, as some are degenerative and may even be terminal, in the case of neoplasms or amyotrophic lateral sclerosis.^19^ |
| Wound located in proximity to the exoskeleton frame | Skin integrity issues that interfere with wearing the exoskeleton may worsen an existing wound with use during RGT. Screening for skin integrity issues is standard screening protocol for Ekso. |
| Severe osteoporosis/-penia as shown with DXA | In a recent systematic review, the incidence of bone fracture at any time during RGT is 3.4% (95% CI: 0.7%–8.1%).^95^ Generally accepted criteria for safe use include ability to stand using an assistive device and absence osteoporosis and existing fractures.^95^ |
| Pre-morbid developmental disability, significant psychological diagnosis, or other cognitive impairment | Identify individuals unable to independently understand instructions – assessed by the Orientation Log (O-LOG), a cognitive assessment tool, to identify the ability for normal orientation.^96^ This tool will be used to exclude cognitively impaired subjects. If the patient does not receive a score of 25 out of 30 or greater, then the patient will not be cleared to consent. |

# **5 STUDY PROCEDURES**

**Duration of Study**

Each patient enrolled in this study will participate in study procedures across their inpatient rehabilitation length of stay commencing with providing informed consent and concluding with discharge from inpatient rehabilitation at BSWIR.

In order to enroll 128 eligible patients with SCI, an estimated 3 years will be necessary to complete all study related activities.

**Visit Windows**

Patients will be asked to complete testing at 2 time points: within one week of admission to inpatient rehabilitation and within one week of discharge from inpatient rehabilitation.

| **Table 6: Patient Timeline** | | | | |
| --- | --- | --- | --- | --- |
| Study Phase | Admission | Treatment (UC or RGT) Intervention | Discharge | Data Source |
| **Consent** | X |  |  |  |
| Demographics | X |  | X | Medical Record |
| **Outcome Testing** |  |  |  |  |
| *Aim 2* |  |  |  |  |
| Walking Score Index for Spinal Cord Injury (WISCI-II) | X |  | X | Medical Record |
| 10-meter walk test (10MWT) | X |  | X | Medical Record |
| Spinal Cord Independence Measure (SCIM) | X |  | X | Medical Record |
| Numeric Pain Rating Scale (NPRS) | X |  | X | Medical Record |
| Fatigue Severity Scale (FSS) | X |  | X | Study Specific |
| Penn Spasm Frequency Scale (PSFS) | X |  | X | Study Specific |
| Patient Health Questionnaire – 9 (PHQ-9) | X |  | X | Study Specific |
| General Anxiety Disorder – 7 (GAD – 7)  International SCI Quality of Life Basic Data Set  Qualitative Questionnaire | X  X |  | X  X  X | Study Specific  Study Specific  Study Specific |
| *Aim 3* |  |  |  |  |
| Heart Rate (HR) |  | X |  | Study Specific |
| Ratings of Perceived Exertion (RPE) |  | X |  | Study Specific |
| Number of steps |  | X |  | Study Specific |

**Screening**

During the screening for consent process, the study staff will utilize the Orientation log (O-LOG), a cognitive assessment tool, to identify the ability for normal orientation. If the patient receives a score of >25 out of 30, the patient will be deemed to have normal orientation necessary for independent consenting and will be cleared to consent.

**Informed Consent**

Consent will be obtained prior to beginning any study-specific intervention. Individuals identified as meeting the inclusion/exclusion criteria during the weekly SCI team rounding meeting will be approached. Research staff and research associates will explain the study and answer any patient questions for clarification. Prospective patients will be approached within the first 7 days of their admission to BSW Rehab and will have until the end of their 10^th^ day to consent to participate. If a patient is not initially appropriate for intervention due to medical reasons and later determined to appropriate during their stay, they may be approached to consent to participate. Research staff and research associates will be responsible for obtaining informed consent. Informed consent will be obtained in private setting, specifically in the patient’s room and with a caregiver, or witness to consenting process if necessary. Qualified patients will be informed about the study and will be given adequate time to decide whether he/she would like to participate in the study. Caregivers of minors will sign consent form and minors will be given an opportunity to assent to project. Patients will be informed that their participation is voluntary and that they can withdraw from the study at any time. Participants will also be encouraged to ask questions throughout their participation in the study.

**Randomization**

Participants meeting the inclusion and exclusion criteria will be approached, screened, consented, and enrolled into the study. After obtaining informed consent, participants will be randomized to either the experimental (RGT) or UC control group using cluster randomization based on specific injury characteristic for stratified block.

The heterogeneity of the population in terms of injury severity is a concern given that these factors are expected to be important to walking recovery. To account for this, patients will be assigned to RGT or UC using 1:1 stratified blocks. Stratification will be based on specific injury characteristics (incomplete tetraplegia and incomplete paraplegia), and within each strata randomization blocks of size 6 will be used to ensure equal distribution between groups.


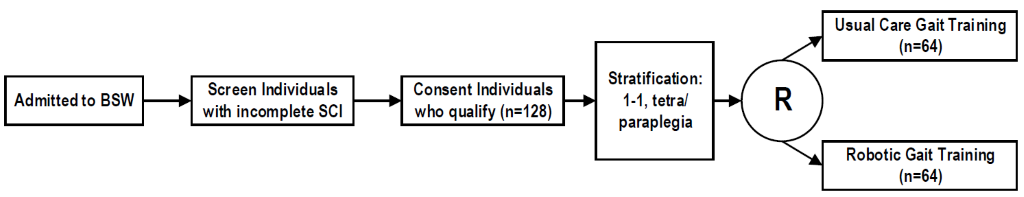


**Study Intervention**

*Staff training*

Therapists will have completed training for delivering RGT with exoskeleton and BSWTT. In addition, the RGT and UC activities will be led by trained therapists who are experienced with treating individuals with SCI. All study staff will be trained on study procedures at the project launch meeting and subsequent support meetings.

*Setting*

All session will be held at Baylor Scott & White Institute of Rehabilitation, an inpatient rehabilitation facility. The location of the intervention activities allows for therapists to provide assistance and supervision at all times.

*Compliance Data Collection*

Participants randomized into the RGT and UC group will be given a Polar heart rate monitor and a pedometer to wear during the gait training sessions. Participants will be asked to return their heart rate monitor be synced to the study staff’s computers and the study staff can review their activity and heart rate reports. Passwords and data will be de-identified and stored in a separate password-protected file. Number of steps will be captured by the Esko device and pedometer for the RGT and UC care group, respectively. **Table 7** provides more detailed information about the devices used in the study.

**Payment**

While we will not pay participants for taking part in study, participants in the UC group will be offered up to 8 sessions of robotic gait training sessions in outpatient rehabilitation for their time/effort to attend each of the sessions and completed assessments which are required for research purposes. Each intervention group will complete a total of 2 assessments.

# **6 STUDY MEASUREMENTS**

**Study Endpoints**

The following data will be collected through the medical record and patient interview at baseline assessment: age; age at injury; injury severity; gender; ethnicity; education level; pre-morbid history of mental illness; residence status; income; insurance type; vocation; and financial status. Level of impairment will be captured using the ASIA Impairment Scale (AIS) to determine injury severity after traumatic SCI by the physician at admission to BSW. The AIS is standard of care at BSW and will be extracted from the medical record. The following data will be collected through medical record and patient interview within one week of admission and discharge: *Gait speed via 10-Meter Walk Test, Spinal Cord Independence Measure (SCIM), Numerical Pain Rating Scale (NPRS), *Fatigue Severity Scale (FSS), Penn Spasm Frequency Scale (PSFS), Patient Health Questionnaire - 9 (PHQ-9), Life Satisfaction Questionnaire (LiSAT-9), Heart Rate (HR), Ratings of Perceived Exertion (RPE), and Number of Steps*. Primary and secondary outcomes for Aim 2 and dose parameters for Aim 3 are described in **Table 7.**

| **Table 7. Outcome Measures for Proposed Project and link to ICF** | | |
| --- | --- | --- |
| **Aim 2 Primary Outcome:** To examine the efficacy of RGT compared to usual care gait training (UC) | | |
| ****Walking Index for Spinal Cord Injury – II (WISCI-II)*** | Our primary outcome, the WISCI-II defines the physical limitation for gait secondary to impairment at the person level and indicates the ability of a person to walk after SCI.^103^ Intrarater and interrater reliability are excellent at 1.0 and 0.98 respectively.^104^ A change of one WISCI level can be considered clinically significant.^105^ | ICF domain = Activity |
| **Aim 2 Secondary Outcomes** | | |
| ****Gait speed via 10-Meter Walk Test (10MWT)*** | Assesses gait speed over a short duration. Gait speed (m/s) is correlated ability to mobility in the community, capacity to perform activities of daily living, risk of falls, re-hospitalization, and risk of cognitive decline.^106^ We will assess gait speed at inpatient rehabilitation admit and discharge, 3- and 6-month follow-up sessions to monitor longitudinal changes throughout the study. For persons with SCI, a combination of the 10MWT and the WISCI-II is recommended to provide the most valid measure of improvement in gait.^107^ A change of >0.06 m/s is considered to exceed minimally clinically important difference (MCID)^108^ and test-retest reliability is excellent (ICC=0.97).^109^ | ICF domain = Activity |
| ****Spinal Cord Independence Measure (SCIM)*** | The SCIM assesses self-care management, respiration and sphincter management, and functional mobility after a SCI. With excellent interrater reliability (r = 0.90),^110^ the SCIM is reported to be more sensitive to functional changes than the FIM.^111^ | ICF domain = Activity |
| ***Numerical Pain Rating Scale (NPRS)*** | Pain is a significant problem in many individuals with SCI. A 0–10 Point Numerical Pain Rating Scale (NRS) is recommended as the outcome measure for pain intensity after SCI^112^ during acute and subacute phases. Pain severity can be categorized into 3 distinct groups as relates to pain interference: 1-3 (mild), 4-7 (moderate), 8-10 (severe)^113^ | ICF domain = Body Structure & Function |
| ****Fatigue Severity Scale (FSS)*** | The Fatigue Severity Scale (FSS) is may be the most widely used measure of fatigue in neurologic disorders^114^ and is a unidimensional measure that measures the effects of fatigue on function. The FSS is easy to use both in clinical practice and research, quick to administer, and its focus on the effects of fatigue on function makes its use in rehabilitation settings particularly appealing.^115^ The FSS has acceptable reliability with regard to internal consistency, test-retest reliability, and validity in persons with SCI.^115^ | ICF domain = Body Structure & Function |
| ***Penn Spasm Frequency Scale (PSFS)*** | The PSFS is a self-report measure to assess a patient’s perception of spasticity frequency and severity following a SCI. With excellent internal consistency (ICC = 0.90),^116^ the current version was modified from the original to include both frequency and severity.^117^ | ICF domain = Body Structure & Function |
| ****Patient Health Questionnaire - 9 (PHQ-9)*** | The PHQ-9 is a self-report measure to assess the presence and intensity of depressive symptoms. For SCI, the PHQ-9 demonstrates excellent internal consistency (Chronbach’s alpha = 0.87)^118^ and construct validity (r = 0.78).^119^ | ICF domain = Body Structure & Function |
| ***General Anxiety Disorder - 7 (GAD - 7)*** | The GAD-7 is a self-report measure to assess severity of anxiety symptom over the past two weeks. When compared with SCI-QOL Anxiety, a correlation of 0.67 and reliability of 0.85 for the GAD-7 provides some support of its use after SCI.^120^ | ICF domain = Body Structure & Function |
| ***International SCI Quality of Life Basic Data Set*** | The ISCIQOL Basic Data Set is a three-item quality of life questionnaire suitable for SCI populations containing 3 variables rating satisfaction with general quality of life, physical health, and psychological health.^121^ Items are answered on a 10-point likert scale that ranges from 0 (completely dissatisfied), to 10 (completely satisfied). | ICF domain = Participation |
| ***Qualitative Questionnaire*** | Participants will be asked questions regarding their experience during the invention such as their likes and dislikes; notable changes or observations; and overall satisfaction with their care. | ICF domain = Participation |
| **Aim 3 Outcomes:** To describe the intensity of RGT and UC gait training | | |
| ****Heart Rate (HR)*** | **Polar heart** **rate monitor** (RS300X, Polar®) will provide data on the day, duration, and intensity (average and maximum) of gait training sessions for both RGT and UC. Participants will be provided a Polar heart rate monitor to wear during each gait training session for the entire length of the study, and gait training session data will be collected weekly. These monitors record beat to beat heart rates and store up to 16 sessions of heart rate data. Each week study staff will upload participants’ heart rate data using Polar’s FlowLink technology via the Polarpersonaltrainer.com website. | ICF domain = Body Structure & Function |
| ****Ratings of Perceived Exertion (RPE)*** | The Borg RPE is a 15-point scale with verbal descriptors to standardize perceived exertion across tasks and individuals. Participants will be asked to provide a self-reported intensity level on the Borg Rating of Perceived Exertion Scale^122^ during RGT and UC gait training sessions. A self-report of 12 to 14 on the RPE indicates moderate intensity. The Borg RPE scale has been shown to be a valid measure of exercise intensity with weighted mean validity coefficient of 0.62 for HR.^123^ | ICF domain = Body Structure & Function |
| ***Number of Steps*** | The Ekso device records several data points for each session including **number of steps**, “Up” time (the amount of time spent standing in the device), “Walk” time (the amount of time spent walking in the device), and device assistance scores. While all of these data values will be recorded to describe each RGT session and tracked to monitor progression of the RGT intervention, the number of steps per session will be utilized as an indicator of RGT session intensity.^42,91^ We will consult our Consumer Advocates for recommendations on incorporating device scores such as “Up” time and “Walk” time as additional measures of intensity.  Number of steps for the UC group will be collected via pedometer during each gait training session. The number of steps captured on the device will be recorded on the CRF after each gait training session. The watch will be reset before each use. | ICF domain = Activity |
| *Each of these measures are core and supplemental instruments of the SCI CDE as developed through the collaboration of the International Spinal Cord Society, the American Spinal Injury Association, and the National Institute of Neurological Disorders and Stroke CDE team, and referenced at http://www.commondataelements.ninds.nih.gov/SCI.aspx. | | |

# **7 STUDY TERMINATION**

Each patient’s participation in the study will be terminated after completion of the study assessments. The end of study date is defined as the date when all data has been collected and statistically analyzed. However, the PI or the IRB may terminate the study at any time.

# **8 DATA QUALITY ASSURANCE**

PI and statistician will be responsible for ensuring the data is accurate. Data management functions conducted in the department will occur on a quarterly basis and will include data quality checks and verification, as well as internal edit and logic checks (e.g., out of range values, internal inconsistencies). Ten percent of charts will be audited for source document and data entry review. Cross tabulation checks using SAS will also be used. Data will be stored and backed-up periodically on the biostatistician’s space on the secure server. Descriptive statistics will be at calculated and included into quarterly reports to ensure the quality of data and progress of the study. In addition, a quality assurance coordinator independent of the study execution will conduct a quality assurance review at beginning of enrollment, midpoint and end of the study.

**Data Safety and Monitoring Board**

A 3-member external Data Safety and Monitoring Board (DSMB), including a Chairperson, will be established for FIRST. The DSMB will monitor the study and review quarterly the following: a) participant recruitment, accrual, retention, and withdrawal information; b) adverse events (AEs) and serious adverse events (SAEs); c) comparison of events that occur between treatment arms; d) individual events of particular concern; e) participant interview and/or performance status outcomes; f) other safety-supporting data requested by the DSMB; and g) summary of protocol violations, completeness and timeliness of study visit data, enrollment eligibility and ineligibility information, noncompliance, and unanticipated problems. None of these individuals on the DSMB will be directly involved in conducting this project or have a stake in its outcome. Funding for the DSMB is included in the budget and includes $300 per member, per year. Each DSMB member will meet at the beginning of the study with the PI and Co-Investigators to learn about the aims of the project, study design, and intervention.

The biostatistician will provide a data report four weeks prior to the DSMB teleconference meeting. Based on these Quarterly reviews, the DSMB may request specific responses from the PI. For example, the PI may be asked to provide additional plans for safety after a severe AE. The DSMB annual reports will enumerate the dates that the Committee meets, and its explicit procedures for monitoring patients’ safety and confidentiality, and data integrity, during the reporting interval. Actions taken by the IRB in response to AE reports will be immediately reported to the Project Officer and Research office.

# **9 STATISTICAL METHODS**

**Sample Size Considerations**

For the proposed project, 128 patients (64 per group) will be enrolled. All analysis will be performed using SAS 9.4 significance set at the 5% level. Sample size calculations were performed to determine the required sample size in each group based on detectable effect size. For 80% power with a 5% significance level we will need to obtain 64 participants in each group to detect a medium effect size of 0.5 and allow for ~10% attrition due to unplanned medical events, acute care transfers, and patient withdrawals. We anticipate minimal attrition as all treatments and outcomes will be assessed during the inpatient rehabilitation stay and we experienced 0% attrition during our feasibility study.

**Study Analysis Methods**

**Aim 1 Analysis.** Our Aim 1 hypothesis is:

Hypothesis 1.1: *We will successfully engage key stakeholders in a community-based participatory research approach to develop the content and structure of the RGT program to accommodate current clinical practice guidelines and integrate emerging robotic exoskeleton technology.*

The Advisory Board of key stakeholders (patients, caregivers, clinicians, researchers, industry members) to review evidence-based literature, advise the research team on the unique aspects and goals of inpatient rehabilitation for people with SCI, review the RGT, and make recommendations for amendments to the RGT program based on our interim and final outcomes of the study over the funding period. The role of the advisory board is necessary to meet the needs of individuals post SCI based on previously demonstrated successful inclusion of RGT into clinical practice during inpatient rehabilitation and detected change in key outcome variables.

**Aim 2 Analysis.** Our Aim 2 hypotheses were:

*Hypothesis 2.1:* *Patients with incomplete SCI who receive RGT will have higher walking function (WISCI-II) at discharge compared to UC.*

*Hypothesis 2.2: Patients with incomplete SCI who receive RGT will have greater improvement of secondary outcomes (gait speed, daily function independence, pain, fatigue, spasm frequency, depressive symptoms, and quality of life) at discharge compared to UC.*

All analysis will be performed using SAS 9.4 with a significance level of 0.05. To determine if patients with incomplete SCI who receive RGT will have higher walking function than UC at discharge (Hypothesis 2.1), WISCI-II scores will be evaluated using a linear model. The distribution of WISCI-II scores will be assessed to determine if a general linear model will be utilized, or if a generalized linear model with an alternative distribution and link function, such as the gamma distribution with a log link, will be more appropriate. Similarly, all measures associated with the secondary outcomes in Hypothesis 2.2 (10MWT, SCIM, NPRS, FSS, PSFS, PHQ-9, LiSAT-9) will be evaluated with general or generalized linear models, as appropriate. A separate model will be run for each outcome. All models for both the primary and secondary outcomes will control for scores at admission as well as the patient’s demographic and impairment information listed in section B.1.i.D.

**Aim 3 Analysis:** Our Aim 3 hypotheses were:

Hypothesis 3.1: *RGT will be of significantly greater intensity with participants (1) spending more minutes per session and total minutes (over all sessions) in moderate intensity exercise (40% to 60% range of heart rate reserve), (2) reporting higher mean RPE across all sessions, and (3) completing more steps during each session and total number of steps over all sessions compared to UC.*

Hypothesis 3.2: *Intensity will be significantly correlated to the primary outcome (WISCI-II) for the RGT and UC groups.*

Hypothesis 3.1 will be evaluated using similar methods as Aim 2. Moderate intensity minutes, average RPE, and steps will be evaluated using individual general or generalized linear models, as appropriate, to determine the association between RGT and UC and intensity. Each model will control for the patient characteristics listed in section B.1.i.D and total number of sessions. Additionally, to determine the relationship between session intensity and walking function (Hypothesis 3.2) correlation coefficients will be calculated between each intensity measure and WISCI-II at admission, discharge, and change score.

For both Aims 2 and 3 stratified subset analysis will be performed for tetraplegia and paraplegia to determine if RGT has a differing impact depending on injury classification.

**Missing Data:** If a patient misses an assessment, their data for that outcome will be treated as missing and *will not* be included in the statistical model. However, the data from the completed assessments for that patient *will* be included. Sensitivity analysis will be performed to assess the impact of missing data, by imputing a missing outcome measure using the overall average change score for the given outcome. The sensitivity analyses will be compared to the initial analysis to determine the impact of missingness on the results

# **11 INVESTIGATOR’S RESPONSIBILITIES**

**Investigator Responsibility**

The principal investigator will be responsible for the conduct of all study activities and safety of participants, including maintaining confidentiality for participants. The principal investigator will also maintain a staff delegation of authority list of appropriately qualified persons to whom the principal investigator has delegated study-related duties. All activities will be performed in accordance with BSWRI policy and federal guidelines.

# **12 ETHICS**

**Institutional Review Board**

The protocol will be reviewed by the Baylor Scott & White Research Institute Institutional Review Board. No study procedures will begin until the principal investigator receives written IRB approval for the protocol, informed consent, and other study documents. The principal investigator will submit continuing review reports on the status of the study. All revisions and events will be reported to the IRB as per federal regulations.

**Ethical Conduct of the Study**

Baylor Scott and White Research Institute has established human subject protection committees that will review and approve all study protocols, informed consent, and documents related to this study. All investigators listed on the project are certified as having completed the Collaborative Institutional Training Initiative human subject protection training and affiliated their credentials with Baylor Scott and White Research Institute. Only study personnel will have access to any personal information. The data collected will not be used to identify individuals and no personally identifiable data will be used in any format (e.g., analysis, presentation, publication, discussion). All participants will be given unique subject IDs and the decoding matrix will be stored on a password-protected Excel spreadsheet on a Baylor computer and will only be available to the principal investigator and delegated study staff. Subject IDs will be used for analysis. Participants will be made aware of efforts to protect their confidentiality.

**Risks and Benefits**

*Physical risks***:** Participants may experience orthostatic hypotension, skin breakdown, falls, fracture, overheating, and autonomic dysreflexia if they have difficulty due to impaired motor and sensory function common to SCI population. All therapy staff is trained to observe and monitor for these risks and methods to limit or resolve these potential concerns.

*Psychological risks***:** Participants may experience frustration during RGT due to motor and sensory deficits common to SCI population. All staff is trained to observe and monitor for frustration and methods to limit or resolve these potential concerns (i.e. institute rest breaks, maintain supervision).

*Social risks***:** No social risks have been identified by participating in this study.

*Legal risks***:** No legal risks have been identified by participating in this study.

*Economic risks*: No economic risks have been identified by participating in this study.

*Direct benefit to research participants*

See below for potential future benefits.

*Potential future benefits to individuals with the condition being studied*

The benefits to participants may include gaining greater walking function and improved health. Study findings could help rehabilitation clinicians identify evidence-based practice walking interventions and facilitate best practice guidelines for patients with SCI.

*Potential benefits to society in general*

The information gained in this study will help validate the importance and benefits of exoskeleton gait training in the outpatient rehabilitation setting for spinal cord injury patients.

*Potential benefits to others involved in research*

Clinicians involved in this research will contribute to evidence-based practice research methods, and facilitation of improvements for best practice for spinal cord injury patients.

*Risk to benefit analysis*

The risks posed to study subjects (e.g. injury while engaging in physical activity, psychological stress associated with treatment) are minimal, while the study poses benefits to the study participants in terms of improved walking recovery after SCI and overall health and function. The research poses potentially great benefits to society, as individuals with SCI and rehabilitation clinicians desire enhanced walking recovery to allow greater independence with mobility for activities and community participation.

It is hoped that the information gained from this proposed study will contribute to an evidence base for a walking retraining intervention with input from key stakeholders that integrates known critical ingredients and established principles of neuroplasticity to specifically address the unique needs of people with incomplete SCI.

# **13 STUDY DOCUMENTATION AND RECORDKEEPING**

In order to assure participant confidentiality, all participants will be assigned a unique study identification number. Only one master list will be maintained by the PI and the study coordinator on a secure network drive in a password protected file. All case report forms and databases will use the subject ID number rather than names or other private health information. Signed consent forms and case report forms will be maintained by the PI behind a locked door in a locked file cabinet.

**Documentation of Data**

All participants will be given a subject identification number. This number will be used on all data collection forms and databases to ensure patient confidentiality. Hospital dates, onset of injury and demographic data will be extracted from patient report or medical record. Other data points can be collected from the participant directly onto the data collection form by the treating clinicians (or a member of the research team). Non-electronic data will be stored in a secured cabinet in the Research Office Suite. Data will then be entered into a secure database that only the study team will be able to access.

**Data Collection and Data Entry into Database**

Research material obtained from study participants will be collected from medical records and assessment and intervention data. All materials will be obtained specifically for research purposes. Members of the research team will collect demographic, physiologic, quantitative observational and/or qualitative information from participants during the study. Demographic, observational and qualitative data will be collected during Aim 2 at admission and discharge inpatient rehabilitation time points at BSW and entered into a secure SAS database by research staff. Physiologic data will be collected during Aim 3 throughout the intervention at BSW. Qualitative/survey data obtained during the admission and discharge time points will be entered into our secure BSW server. All demographic, observational, and physiologic data will be recorded on paper source documents and later entered to either REDCap or SAS database by research staff. All data will be stored on a secure server, with security meeting BSWRI standards.

Data will be collected via our medical record (Epic), patient interviews, and testing conducted by the study coordinators. Patient testing for study specific patient-reported outcomes will be conducted in the research lab, located in the inpatient rehabilitation hospital. We anticipate study specific assessments to require 1 hour/assessment. Clinical measures and measures of intervention intensity (e.g., HR, RPE) will be assessed by the treating physical therapists during therapy sessions as part of usual care. The outcome assessment schedule is depicted in **Table 7**.

The following data will be collected through the medical record and patient interview: age; age at injury; injury severity; gender; ethnicity; education level; pre-morbid history of mental illness; residence status; income; insurance type; vocation; and financial status. Level of impairment will be captured using the ASIA Impairment Scale (AIS) to determine injury severity after traumatic SCI by the physician at admission to BSW. The AIS is standard of care at BSW and will be extracted from the medical record.

**Record Retention**

All paper source documents will be kept in a double locked storage cabinet in the BSWIR research office. All electronic data will be kept on a secure server per federal guidelines. All data will be maintained for two years after study termination per federal guidelines and will be disposed of in accordance to current BSWRI policy.

**Protocol Deviations**

All protocol deviations will be recorded in the participant’s chart and regulatory binder as they occur, and subsequently formally to the IRB at continuing review

**Management of Subject Safety/ Adverse events**

The study staff (PI, Co-Investigators, research coordinators and assistants) will be responsible for collecting and recording all clinical data. As results are collected, all Adverse Events will be identified, graded for severity and assigned causality. **Only those adverse events directly related or caused by the study will be reported to the IRB and compiled for periodic review**. After assigning causality, the PI will decide the course of action for the study participant. The PI will evaluate all Adverse Events and determine whether the Adverse Event affects the risk/benefit ratio of the study and whether modifications to the protocol or informed consent form are required*.* Throughout this process, the PI will inform and collaborate with the research team.

The plan to monitor participant data and safety will specifically include the following: (1) Dr. Swank (PI) will inspect collected data; (2) The IRB offices will be contacted if there is an Adverse Event due to participation; (3) the research protocol will be revised if it is determined that the protocol or intervention presents an unforeseen risk to participants; (4) if an event occurs that requires immediate attention and Dr. Swank is unavailable, then members of the research team will follow the emergency procedures put in place by the research team, which may include calling emergency medical services.

# **14 USE OF INFORMATION AND PUBLICATION**

The information gathered by this study may be used in a research report and submitted to multiple scholarly journals for publication. The investigators may also present their findings on a poster at a professional conference. No information on the participants’ identities will be included in the report.

# **15 REFERENCES**

1.del-Ama AJ, Gil-Agudo Á, Pons JL, Moreno JC. Hybrid gait training with an overground robot for people with incomplete spinal cord injury: a pilot study. Frontiers in human neuroscience. 2014;8.

2.Lajeunesse V, Vincent C, Routhier F, Careau E, Michaud F. Exoskeletons' design and usefulness evidence according to a systematic review of lower limb exoskeletons used for functional mobility by people with spinal cord injury. Disability and Rehabilitation: Assistive Technology. 2016;11(7):535.

3.Holanda LJ, Silva PM, Amorim TC, Lacerda MO, Simão CR, Morya E. Robotic assisted gait as a tool for rehabilitation of individuals with spinal cord injury: a systematic review. Journal of neuroengineering and rehabilitation. 2017;14(1):126.

4.Mekki M, Delgado AD, Fry A, Putrino D, Huang V. Robotic Rehabilitation and Spinal Cord Injury: a Narrative Review. Neurotherapeutics. 2018:1-14.

5.Heinemann AW, Jayaraman A, Mummidisetty CK, et al. Experience of Robotic Exoskeleton Use at Four Spinal Cord Injury Model Systems Centers. Journal of Neurologic Physical Therapy. 2018;42(4):256-267.

6.Tsai C-Y, Delgado AD, Weinrauch WJ, et al. Exoskeletal-Assisted Walking during Acute Inpatient Rehabilitation Leads to Motor and Functional Improvement in Persons with Spinal Cord Injury–a Pilot Study. 2019.

7.Behrman AL, Bowden MG, Nair PM. Neuroplasticity after spinal cord injury and training: an emerging paradigm shift in rehabilitation and walking recovery. Physical Therapy. 2006;86(10):1406.

8.Consortium for Spinal Cord M. Outcomes following traumatic spinal cord injury: clinical practice guidelines for health-care professionals. Consortium for Spinal Cord Medicine; 1999.

9.Swank C, Sikka S, Driver S, Bennett M, Callender L. Feasibility of integrating robotic exoskeleton gait training in inpatient rehabilitation. Disability and Rehabilitation: Assistive Technology. 2019:1-9.

10.Arnold D, Swank C, Sikka S, Bennett M, Callender L, Driver S. Robotic Gait Training for Spinal Cord Injury During Inpatient Rehabilitation – retrospective findings. Paper presented at: Academy of Spinal Cord Injury Professionals2019; Nashville, TN.

11.Swank C, Trammell M, Bennett M, et al. Robotic Gait Training During Inpatient Rehabilitation – retrospective findings. Archives of Physical Medicine & Rehabilitation. 2019.

12.Swank C, DiPasquale J, Sikka S. Physiologic response to Robotic Gait Training during Inpatient Rehabilitation. In: Baylor Scott & White Institute for Rehabilitation; 2019.

13.Swank C, Wang-Price S, Gao F, Almutairi S, Bednarz H, Seeber K. Muscle Activity During Gait With A Robotic Exoskeleton In People With Spinal Cord Injury. Paper presented at: Combined Sections Meeting of American Physical Therapy Association2019; Washington DC.

14.Swank C, Wang-Price S, Gao F, Almutairi S, Seeber K, Bednarz H. Gait Kinematics in People with Incomplete Spinal Cord Injury with a Robotic Exoskeleton. Paper presented at: Combined Sections Meeting of American Physical Therapy Association2019; Washington DC.

15.National Spinal Cord Injury Statistical Center. Facts and Figures at a Glance. 2018; https://www.nscisc.uab.edu, 2018.

16.Savic G, DeVivo MJ, Frankel HL, Jamous MA, Soni BM, Charlifue S. Long-term survival after traumatic spinal cord injury: a 70-year British study. Spinal cord. 2017;55(7):651-658.

17.Shavelle RM, DeVivo MJ, Brooks JC, Strauss DJ, Paculdo DR. Improvements in long-term survival after spinal cord injury? Arch Phys Med Rehabil. 2015;96(4):645-651.

18.Dijkers MJSc. Quality of life after spinal cord injury: a meta analysis of the effects of disablement components. 1997;35(12):829.

19.New PW, Eriks-Hoogland I, Scivoletto G, et al. Important Clinical Rehabilitation Principles Unique to People with Non-traumatic Spinal Cord Dysfunction. Topics in spinal cord injury rehabilitation. 2017;23(4):299-312.

20.Sekhon LH, Fehlings MG. Epidemiology, demographics, and pathophysiology of acute spinal cord injury. Spine. 2001;26(24S):S2-S12.

21.Fleerkotte BM, Koopman B, Buurke JH, van Asseldonk EH, van der Kooij H, Rietman JS. The effect of impedance-controlled robotic gait training on walking ability and quality in individuals with chronic incomplete spinal cord injury: an explorative study. Journal of neuroengineering and rehabilitation. 2014;11(1):1.

22.van Middendorp JJ, Hosman AJF, Van de Meent H. Who wants to walk? Preferences for recovery after SCI: a longitudinal and cross-sectional study. Spinal Cord. 2009;47(3):268.

23.Bogey R, George Hornby T. Gait training strategies utilized in poststroke rehabilitation: are we really making a difference? Topics in Stroke Rehabilitation. 2007;14(6):1.

24.Buzzelli S, Di Francesco L, Villani S, Giaquinto S. Gait in stabilized hemiplegic outpatients in rehabilitation. Disability and rehabilitation. 2003;25(17):964-967.

25.Saadat S, Javadi M, Divshali BS, et al. Health-related quality of life among individuals with long-standing spinal cord injury: a comparative study of veterans and non-veterans. BMC Public Health. 2010;10(1):6.

26.Semerjian T, Montague S, Dominguez J, Davidian A, de Leon R. Enhancement of quality of life and body satisfaction through the use of adapted exercise devices for individuals with spinal cord injuries. Topics in spinal cord injury rehabilitation. 2005;11(2):95-108.

27.Hicks AL, Adams MM, Ginis KM, et al. Long-term body-weight-supported treadmill training and subsequent follow-up in persons with chronic SCI: effects on functional walking ability and measures of subjective well-being. Spinal Cord. 2005;43(5):291.

28.Ditunno P, Patrick M, Stineman M, Ditunno J. Who wants to walk? Preferences for recovery after SCI: a longitudinal and cross-sectional study. Spinal cord. 2008;46(7):500-506.

29.Gomes-Osman J, Cortes M, Guest J, Pascual-Leone A. A systematic review of experimental strategies aimed at improving motor function after acute and chronic spinal cord injury. Journal of neurotrauma. 2016;33(5):425-438.

30.Dobkin B, Apple D, Barbeau H, et al. Weight-supported treadmill vs over-ground training for walking after acute incomplete SCI. Neurology. 2006;66(4):484-493.

31.Wessels M, Lucas C, Eriks I, de Groot S. Body weight-supported gait training for restoration of walking in people with an incomplete spinal cord injury: a systematic review. Journal of rehabilitation medicine. 2010;42(6):513-519.

32.Mehrholz J, Harvey L, Thomas S, Elsner B. Is body-weight-supported treadmill training or robotic-assisted gait training superior to overground gait training and other forms of physiotherapy in people with spinal cord injury? A systematic review. Spinal cord. 2017;55(8):722.

33.Harvey L, Glinsky J, Bowden J. The effectiveness of 22 commonly administered physiotherapy interventions for people with spinal cord injury: a systematic review. Spinal cord. 2016;54(11):914.

34.Field-Fote EC, Roach KE. Influence of a locomotor training approach on walking speed and distance in people with chronic spinal cord injury: a randomized clinical trial. Physical therapy. 2011;91(1):48-60.

35.Lam T, Pauhl K, Ferguson A, Malik RN, Krassioukov A, Eng JJ. Training with robot-applied resistance in people with motor-incomplete spinal cord injury: Pilot study. JRehabilResDev. 2015;52(1).

36.Field-Fote EC, Yang JF, Basso DM, Gorassini MA. Supraspinal Control Predicts Locomotor Function and Forecasts Responsiveness to Training after Spinal Cord Injury. J Neurotrauma. 2017;34(9):1813-1825.

37.van der Scheer JW, Ginis KAM, Ditor DS, et al. Effects of exercise on fitness and health of adults with spinal cord injury: A systematic review. Neurology. 2017;89(7):736-745.

38.Cramer SC, Sur M, Dobkin BH, et al. Harnessing neuroplasticity for clinical applications. Brain. 2011;134(6):1591-1609.

39.El-Sayes J, Harasym D, Turco CV, Locke MB, Nelson AJ. Exercise-Induced Neuroplasticity: A Mechanistic Model and Prospects for Promoting Plasticity. The Neuroscientist. 2018:1073858418771538.

40.Loy K, Schmalz A, Hoche T, et al. Enhanced voluntary exercise improves functional recovery following spinal cord injury by impacting the local neuroglial injury response and supporting the rewiring of supraspinal circuits. Journal of Neurotrauma. 2018;35(24):2904-2915.

41.Vega SR, Abel T, Lindschulten R, Hollmann W, Bloch W, Strüder H. Impact of exercise on neuroplasticity-related proteins in spinal cord injured humans. Neuroscience. 2008;153(4):1064-1070.

42.Yang JF, Musselman KE, Livingstone D, et al. Repetitive mass practice or focused precise practice for retraining walking after incomplete spinal cord injury? A pilot randomized clinical trial. Neurorehabilitation and neural repair. 2014;28(4):314-324.

43.Hubbard IJ, Parsons MW, Neilson C, Carey LMJOti. Task‐specific training: evidence for and translation to clinical practice. 2009;16(3‐4):175-189.

44.Esquenazi A, Talaty M, Jayaraman A. Powered Exoskeletons for Walking Assistance in Persons with Central Nervous System Injuries: A Narrative Review. Pm&R. 2016.

45.Miller LE, Zimmermann AK, Herbert WG. Clinical effectiveness and safety of powered exoskeleton-assisted walking in patients with spinal cord injury: systematic review with meta-analysis. Medical Devices (Auckland, NZ). 2016;9:455.

46.Escalona MJ, Brosseau R, Vermette M, et al. Cardiorespiratory demand and rate of perceived exertion during overground walking with a robotic exoskeleton in long-term manual wheelchair users with chronic spinal cord injury: A cross-sectional study. Ann Phys Rehabil Med. 2018.

47.Dietz V, Fouad K. Restoration of sensorimotor functions after spinal cord injury. Brain. 2014;137(Pt 3):654.

48.Kleim J, et al. BDNF vall66met polymorphism is associated with modified experience-dependent plasticity in human motor cortex. Nat Neurosci. 2006;9:735.

49.Bauer UE, Briss PA, Goodman RA, Bowman BA. Prevention of chronic disease in the 21st century: elimination of the leading preventable causes of premature death and disability in the USA. Lancet (London, England). 2014;384(9937):45-52.

50.Centers for Disease C, Prevention. State-specific prevalence of obesity among adults with disabilities--eight states and the District of Columbia, 1998-1999. MMWRMorbidity and mortality weekly report. 2002;51(36):805-808.

51.Armour BS, Campbell VA, Crews JE, Malarcher A, Maurice E, Richard RA. State-level prevalence of cigarette smoking and treatment advice, by disability status, United States, 2004. Preventing chronic disease. 2007;4(4):A86.

52.Froehlich-Grobe K, Lee J, Washburn RA. Disparities in obesity and related conditions among Americans with disabilities. American Journal of Preventive Medicine. 2013;45(1):83-90.

53.Weil E, Wachterman M, McCarthy EP, et al. Obesity among adults with disabling conditions. JAMA : the journal of the American Medical Association. 2002;288(10):1265-1268.

54.Rasch EK, Hochberg MC, Magder L, Magaziner J, Altman BM. Health of community-dwelling adults with mobility limitations in the United States: prevalent health conditions. Part I. Archives of Physical Medicine and Rehabilitation. 2008;89(2):210-218.

55.Rasch EK, Magder L, Hochberg MC, Magaziner J, Altman BM. Health of community-dwelling adults with mobility limitations in the United States: incidence of secondary health conditions. Part II. Archives of Physical Medicine and Rehabilitation. 2008;89(2):219-230.

56.National Institute on D, Rehabilitation R. National Institute on Disability and Rehabilitation Research; Long-Range Plan for Fiscal Years 2013-2017. Washington DC: Federal Registrar;2013.

57.Carmona RH, Giannini M, Bergmark B, Cabe J. The Surgeon General's Call to Action to Improve the Health and Wellness of Persons with Disabilities: historical review, rationale, and implications 5 years after publication. Disability and health journal. 2010;3(4):229-232.

58.Koh HK. A 2020 vision for healthy people. New England Journal of Medicine. 2010;362(18):1653-1656.

59.Medical Rehabilitation Coordinating Committee N. National Institutes of Health research plan on rehabilitation. Assistive Technology. 2017;29(2):106-109.

60.Buurke JH, Nene AV, Kwakkel G, Erren-Wolters V, Ijzerman MJ, Hermens HJ. Recovery of gait after stroke: what changes? NeurorehabilNeural Repair. 2008;22(6):676.

61.Rossi P, Lipsey M, Freeman HJEAsa. Expressing and assessing program theory. 2004;7:133-168.

62.Kirshblum SC, Burns SP, Biering-Sorensen F, et al. International standards for neurological classification of spinal cord injury (revised 2011). The journal of spinal cord medicine. 2011;34(6):535-546.

63.New PW, Sundararajan V. Incidence of non-traumatic spinal cord injury in Victoria, Australia: a population-based study and literature review. Spinal cord. 2008;46(6):406-411.

64.Noonan VK, Fingas M, Farry A, et al. Incidence and prevalence of spinal cord injury in Canada: a national perspective. Neuroepidemiology. 2012;38(4):219-226.

65.Fehlings MG, Tetreault LA, Aarabi B, et al. A clinical practice guideline for the management of patients with acute spinal cord injury: recommendations on the type and timing of rehabilitation. Global spine journal. 2017;7(3_suppl):231S-238S.

66.Chen G, Chan CK, Guo Z, Yu H. A review of lower extremity assistive robotic exoskeletons in rehabilitation therapy. Critical reviews in biomedical engineering. 2013;41(4-5):343-363.

67.Sale P, Franceschini M, Waldner A, Hesse S. Use of the robot assisted gait therapy in rehabilitation of patients with stroke and spinal cord injury. European journal of physical and rehabilitation medicine. 2012;48(1):111-121.

68.Tefertiller C, Hays K, Jones J, et al. Initial Outcomes from a Multicenter Study Utilizing the Indego Powered Exoskeleton in Spinal Cord Injury. Topics in spinal cord injury rehabilitation. 2017;24(1):78-85.

69.Louie DR, Eng JJ, Lam T. Gait speed using powered robotic exoskeletons after spinal cord injury: a systematic review and correlational study. Journal of neuroengineering and rehabilitation. 2015;12(1):1.

70.Zeilig G, Weingarden H, Zwecker M, Dudkiewicz I, Bloch A, Esquenazi A. Safety and tolerance of the ReWalk™ exoskeleton suit for ambulation by people with complete spinal cord injury: A pilot study. JSpinal Cord Med. 2012;35(2):96.

71.Kolakowsky-Hayner SA, Crew J, Moran S, Shah A. Safety and feasibility of using the EksoTM bionic exoskeleton to aid ambulation after spinal cord injury. Journal of Spine. 2013;2013.

72.Esquenazi A, Talaty M, Packel A, Saulino M. The ReWalk powered exoskeleton to restore ambulatory function to individuals with thoracic-level motor-complete spinal cord injury. American journal of physical medicine & rehabilitation. 2012;91(11):911-921.

73.Wall A, Borg J, Palmcrantz S, Gutierrez-Farewik E. Is there any added benefit of using a hybrid robotic exoskeleton for gait training in early stroke rehabilitation? Gait Posture. 2016;49:125.

74.Chang S-H, Afzal T, Berliner J, Francisco GE. Exoskeleton-assisted gait training to improve gait in individuals with spinal cord injury: a pilot randomized study. Pilot and feasibility studies. 2018;4(1):62.

75.Baunsgaard CB, Nissen UV, Brust AK, et al. Exoskeleton gait training after spinal cord injury: An exploratory study on secondary health conditions. J Rehabil Med. 2018;50(9):806-813.

76.Karelis AD, Carvalho LP, Castillo MJ, Gagnon DH, Aubertin-Leheudre M. Effect on body composition and bone mineral density of walking with a robotic exoskeleton in adults with chronic spinal cord injury. Journal of rehabilitation medicine : official journal of the UEMS European Board of Physical and Rehabilitation Medicine. 2017;49(1):84-87.

77.Chisholm AE, Alamro RA, Williams AM, Lam T. Overground vs. treadmill-based robotic gait training to improve seated balance in people with motor-complete spinal cord injury: a case report. J Neuroeng Rehabil. 2017;14(1):27.

78.Asselin P, Knezevic S, Kornfeld S, et al. Heart rate and oxygen demand of powered exoskeleton-assisted walking in persons with paraplegia. J Rehabil Res Dev. 2015;52(2):147-158.

79.Evans N, Hartigan C, Kandilakis C, Pharo E, Clesson I. Acute Cardiorespiratory and Metabolic Responses During Exoskeleton-Assisted Walking Overground Among Persons with Chronic Spinal Cord Injury. Topics in spinal cord injury rehabilitation. 2015;21(2):122-132.

80.Shin JC, Kim JY, Park HK, Kim NY. Effect of Robotic-Assisted Gait Training in Patients With Incomplete Spinal Cord Injury. Annals of rehabilitation medicine. 2014;38(6):719.

81.Swank C, Galvan C, DiPasquale J, Callender L, Sikka S, Driver S. Lessons Learned from Robotic Gait Training during Rehabilitation – therapeutic & medical severity considerations over 3 years. American journal of physical medicine & rehabilitation. 2019.

82.Swank C, Galvan C, DiPasquale J, Callender L, Sikka S, Driver S. Lessons Learned from Robotic Gait Training during Rehabilitation – therapeutic & medical severity considerations over 3 years. Paper presented at: American Congress of Rehabilitation Medicine.2019; Chicago, IL.

83.Swank C, Wang-Price, S., Almutairi, S., & Gao, F.,. Walking in a Robotoic Exoskeleton Does Not Mimic Natural Gait. JMIR Rehabilitation and Assistive. 2018;In Press.

84.Breslin A, Lovotti A, Swank C, Almutairi S, Wang-Price S, Gao F. Muscle Activity During Gait in People with Stroke before and after use of Robotic Exoskeleton. Paper presented at: Combined Sections Meeting of American Physical Therapy Association2020; Denver, CO.

85.Crozier J, Roig M, Eng JJ, et al. High-Intensity Interval Training After Stroke: An Opportunity to Promote Functional Recovery, Cardiovascular Health, and Neuroplasticity. Neurorehabilitation and neural repair. 2018:1545968318766663.

86.Landers MR, Navalta JW, Murtishaw AS, Kinney JW, Richardson SP. A high-intensity exercise boot camp for persons with Parkinson disease: a Phase II, pragmatic, randomized clinical trial of feasibility, safety, signal of efficacy, and disease mechanisms. Journal of Neurologic Physical Therapy. 2019;43(1):12-25.

87.Lin T-W, Tsai S-F, Kuo Y-M. Physical exercise enhances neuroplasticity and delays Alzheimer’s disease. Brain Plasticity. 2018(Preprint):1-16.

88.Ginis KAM, van der Scheer JW, Latimer-Cheung AE, et al. Evidence-based scientific exercise guidelines for adults with spinal cord injury: an update and a new guideline. Spinal cord. 2018;56(4):308.

89.Physical Activity Guidelines Advisory C. Physical activity guidelines for Americans. Washington, DC: US Department of Health and Human Services. 2008:15.

90.Goosey-Tolfrey V, Lenton J, Goddard J, Oldfield V, Tolfrey K, Eston R. Regulating intensity using perceived exertion in spinal cord-injured participants. Medicine & Science in Sports & Exercise. 2010;42(3):608-613.

91.Holleran CL, Hennessey PW, Leddy AL, et al. High-Intensity Variable Stepping Training in Patients With Motor Incomplete Spinal Cord Injury: A Case Series. Journal of Neurologic Physical Therapy. 2018;42(2):94-101.

92.Centers for Medicare & Medicaid Services. Inpatient Rehabilitation Facilities. 2012; https://www.cms.gov/Medicare/Provider-Enrollment-and-Certification/CertificationandComplianc/InpatientRehab.html. Accessed January 28, 2019, 2019.

93.Paralyzed Veterans of America. Publications: Clinical Practice Guidelines. 2018; https://www.pva.org/publications/clinical-practice-guidelines. Accessed October 13, 2018.

94.Gad P, Gerasimenko Y, Zdunowski S, et al. Weight bearing over-ground stepping in an exoskeleton with non-invasive spinal cord neuromodulation after motor complete paraplegia. Frontiers in neuroscience. 2017;11:333.

95.Miller LE, Zimmermann AK, Herbert WG. Clinical effectiveness and safety of powered exoskeleton-assisted walking in patients with spinal cord injury: systematic review with meta-analysis. Medical devices (Auckland, NZ). 2016;9:455.

96. Jackson WT, Novack TA, Dowler RN. Effective serial measurement of cognitive orientation in rehabilitation: the Orientation Log. Archives of physical medicine and rehabilitation. 1998 Jun 1;79(6):718-21.

97.Benson I, Hart K, Tussler D, van Middendorp JJ. Lower-limb exoskeletons for individuals with chronic spinal cord injury: Findings from a feasibility study. Clinical rehabilitation. 2015:0269215515575166.

98.White GW, Nary DE, Froehlich AK. Consumers as Collaborators in Research and Action. Journal of Prevention & Intervention in the Community. 2001;21(2):15.

99.Drum CE, Peterson JJ, Culley C, et al. Guidelines and criteria for the implementation of community-based health promotion programs for individuals with disabilities. American Journal of Health Promotion. 2009;24(2):93-101.

100.Driver S, Reynolds, M., Kramer, K.,. Modifying an evidence-based lifestyle program for individuals with traumatic brain injury. Brain Injury. 2017;Article In Press.

101.Driver S, Swank C, Bailey R, et al. Adapting An Evidence-Based Healthy Lifestyle Program For People After Stroke. 2019;100(10):e41.

102.Betts AC, Froehlich-Grobe K, Driver S, Carlton D, Kramer MK. Reducing barriers to healthy weight: Planned and responsive adaptations to a lifestyle intervention to serve people with impaired mobility. Disability and health journal. 2018;11(2):315-323.

103.Ditunno PL, Dittuno JF. Walking index for spinal cord injury (WISCI II): scale revision. Spinal Cord. 2001;39(12):654.

104.Marino RJ, Scivoletto G, Patrick M, et al. Walking index for spinal cord injury version 2 (WISCI-II) with repeatability of the 10-m walk time: Inter- and intrarater reliabilities. AmJPhysMedRehabil. 2010;89(1):7.

105.Burns AS, Delparte JJ, Patrick M, Marino RJ, Ditunno JF. The reproducibility and convergent validity of the walking index for spinal cord injury (WISCI) in chronic spinal cord injury. NeurorehabilNeural Repair. 2011;25(2):149.

106.Middleton A, Fritz SL, Lusardi M. Walking speed: the functional vital sign. Journal of Aging and Physical Activity. 2015;23(2):314-322.

107.Jackson AB, Carnel CT, Ditunno JF, et al. Outcome measures for gait and ambulation in the spinal cord injury population. The journal of spinal cord medicine. 2008;31(5):487-499.

108.Lam T, Noonan VK, Eng JJ. A systematic review of functional ambulation outcome measures in spinal cord injury. Spinal Cord. 2008;46(4):246.

109.Bowden MG, Behrman AL. Step Activity Monitor: accuracy and test-retest reliability in persons with incomplete spinal cord injury. J Rehabil Res Dev. 2007;44(3):355-362.

110.Itzkovich M, Tamir A, Philo O, et al. Reliability of the Catz-Itzkovich Spinal Cord Independence Measure assessment by interview and comparison with observation. American journal of physical medicine & rehabilitation. 2003;82(4):267.

111.Catz A, Itzkovich M, Agranov E, Ring H, Tamir A. SCIM-spinal cord independence measure: a new disability scale for patients with spinal cord lesions. Spinal Cord. 1997;35(12):850.

112.Bryce TN, Budh CN, Cardenas DD, et al. Pain after spinal cord injury: an evidence-based review for clinical practice and research. Report of the National Institute on Disability and Rehabilitation Research Spinal Cord Injury Measures meeting. The journal of spinal cord medicine. 2007;30(5):421-440.

113.Hanley MA, Masedo A, Jensen MP, Cardenas D, Turner JA. Pain interference in persons with spinal cord injury: classification of mild, moderate, and severe pain. The journal of pain : official journal of the American Pain Society. 2006;7(2):129-133.

114.Dittner AJ, Wessely SC, Brown RG. The assessment of fatigue: a practical guide for clinicians and researchers. Journal of psychosomatic research. 2004;56(2):157-170.

115.Anton HA, Miller WC, Townson AF. Measuring fatigue in persons with spinal cord injury. Arch Phys Med Rehabil. 2008;89(3):538-542.

116.Hsieh J, Wolfe D, Miller W, Curt AJSc. Spasticity outcome measures in spinal cord injury: psychometric properties and clinical utility. 2008;46(2):86.

117.Priebe MM, Sherwood AM, Thornby JI, Kharas NF, Markowski JJAopm, rehabilitation. Clinical assessment of spasticity in spinal cord injury: a multidimensional problem. 1996;77(7):713-716.

118.Bombardier CH, Richards JS, Krause JS, Tulsky D, Tate DGJAopm, rehabilitation. Symptoms of major depression in people with spinal cord injury: implications for screening. 2004;85(11):1749-1756.

119.Krause JS, Saunders LL, Reed KS, Coker J, Zhai Y, Johnson EJRp. Comparison of the Patient Health Questionnaire and the Older Adult Health and Mood Questionnaire for self-reported depressive symptoms after spinal cord injury. 2009;54(4):440.

120.Kisala PA, Tulsky DS, Kalpakjian CZ, Heinemann AW, Pohlig RT, Carle A, & Choi SW. Measuring anxiety after spinal cord injury: Development and psychometric characteristics of the SCI-QOL Anxiety item bank and linkage with GAD-7. J Spinal Cord Med. 2015;38(3):315-325

121.Charlifue S, Post MW, Biering-Sørensen F, Catz A, Dijkers M, Geyh S, Horsewell J, Noonan V, Noreau L, Tate D, Sinnott KA. International spinal cord injury quality of life basic data set. Spinal Cord. 2012;50(9):672-5.

122.Borg GA. Psychophysical bases of perceived exertion. MedSciSports Exerc. 1982;14(5):377.

123.Chen MJ, Fan X, Moe ST. Criterion-related validity of the Borg ratings of perceived exertion scale in healthy individuals: a meta-analysis. Journal of sports sciences. 2002;20(11):873-899.

124.Mowbray CT, Holter MC, Teague GB, Bybee D. Fidelity criteria: Development, measurement, and validation. American journal of evaluation. 2003;24(3):315-340.
